# Enzymes, Reacting with Organophosphorus Compounds as Detoxifiers: Diversity and Functions

**DOI:** 10.3390/ijms22041761

**Published:** 2021-02-10

**Authors:** Ilya Lyagin, Elena Efremenko

**Affiliations:** Faculty of Chemistry, Lomonosov Moscow State University, Lenin Hills 1/3, 119991 Moscow, Russia; lyagin@mail.ru

**Keywords:** organophosphorus toxin, hydrolase, cholinesterase, paraoxonase, phosphatase, senescence marker protein 30, catalysis mechanism, active center, docking, computational modeling

## Abstract

Organophosphorus compounds (OPCs) are able to interact with various biological targets in living organisms, including enzymes. The binding of OPCs to enzymes does not always lead to negative consequences for the body itself, since there are a lot of natural biocatalysts that can catalyze the chemical transformations of the OPCs via hydrolysis or oxidation/reduction and thereby provide their detoxification. Some of these enzymes, their structural differences and identity, mechanisms, and specificity of catalytic action are discussed in this work, including results of computational modeling. Phylogenetic analysis of these diverse enzymes was specially realized for this review to emphasize a great area for future development(s) and applications.

## 1. Introduction

Organophosphorus compounds (OPCs) are extremely dangerous and have a wide variety of chemical structures mainly presented by phosphotriester, thiophosphotriester, and phosphorothioester moieties [1]. Pesticides currently approved for application among OPCs contain sulfur instead of phosphoryl oxygen. Such substitution is believed to reduce the level of acute toxicity of these compounds to humans. Unlike pesticides, all chemical warfare agents belonging to OPCs are chiral phosphonates with a C–P bond. The most famous nerve agents among them are sarin and soman, both containing a labile fluoride group, and V-gases, containing branched alkylthiols.

The main hazard of OPCs to living beings is their reactivity towards Ser residue which is present in active centers of numerous enzymes [2]. For example, acetylcholinesterase necessary for the normal transmission of nervous signals is completely and rapidly inhibited in an irreversible manner by the most OPCs. Other serine hydrolases are also affected [3]. Besides, OPCs can randomly bind to other proteins [4], proteinaceous receptors [5], etc. That totally leads to multiple dysfunctions of cellular regulation [6], oxidative stress, and apoptosis [7]. To avoid these negative outcomes, biocatalytic detoxification of OPCs seems to be the most reliable.

**Figure 1:**
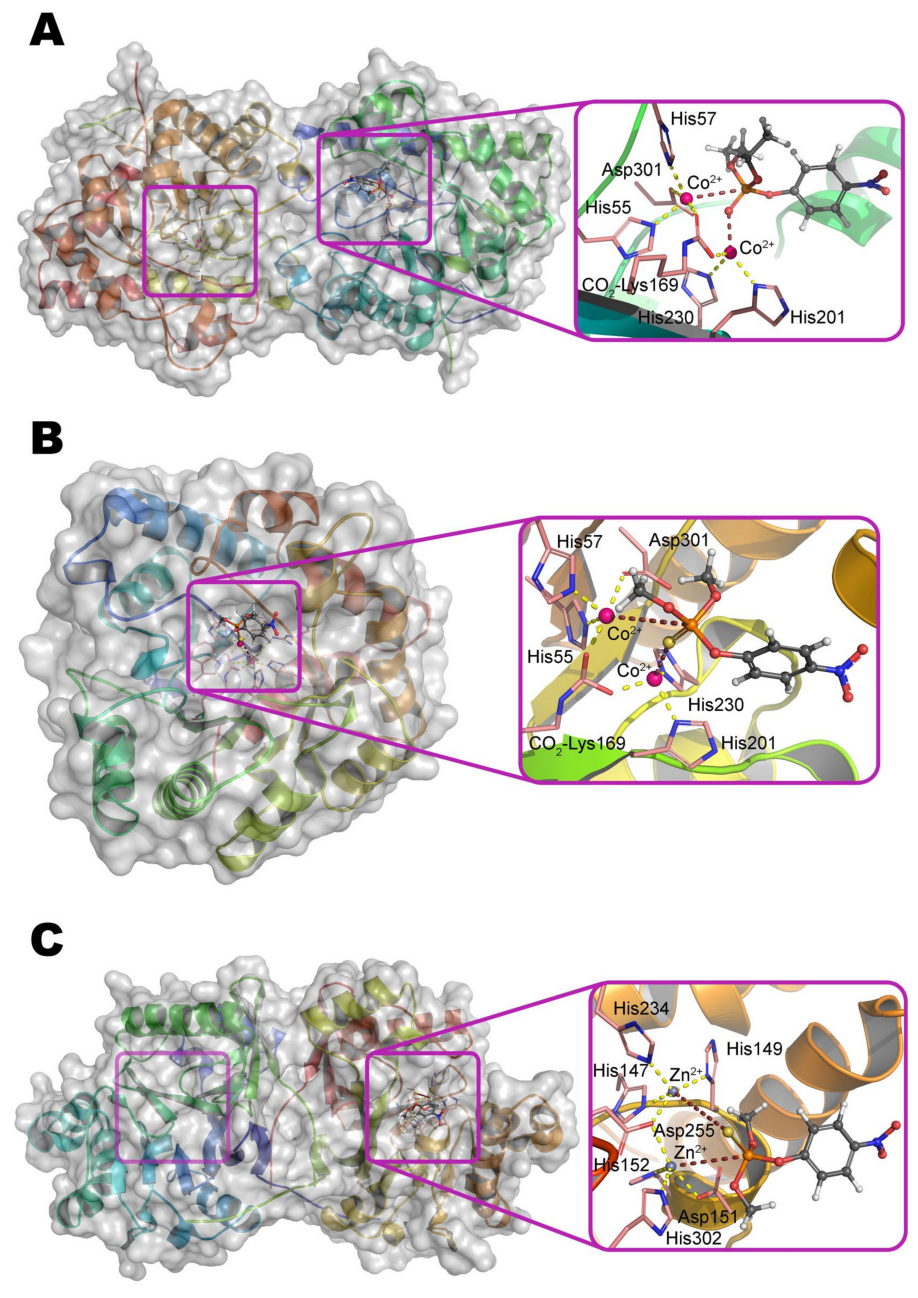
The structure of OPH dimer (**A**), OpdA monomer (**B**), and MPH dimer (**C**) according to X-ray diffraction data (PDB 1QW7, 2D2G, and 1P9E, respectively). The entrance to the active centers of the enzymes is framed, and their structures are shown in detail within insets. The binding of the substrate paraoxon (**A**) and methyl parathion (**B**,**C**) was determined by molecular docking using AutoDock Vina, as described previously [11] (Appendix A). The coordination bonds of metal ions with amino acid residues in active centers, as well as with labile groups of the substrate are shown by a dashed line.

Despite the structural diversity of OPCs, a large number of enzymes have been discovered to be catalytically active with these compounds. Most of these enzymes (organophosphorus hydrolase, OPH; methyl parathion hydrolase, MPH; organophosphorus acid anhydrolase, OPAA; etc.) belong to hydrolases (Figure 1), but there are oxidoreductases and lyases also, and some representative examples of these enzyme classes will be discussed later. Various pro- and eukaryotic organisms are sources of these enzymes, and new ones are constantly discovered. Over the past decades, the catalytic characteristics of these enzymes, as well as crystallographic structures of many of them, have been determined, and the mechanisms of their action have been explained.

The purpose of this work was to review and consolidate current literature data about enzymes being catalytically active with OPCs, to emphasize modern approaches to OPCs’ detoxification, applicability of different enzymes for this purpose, and for investigation of their catalytic mechanisms particularly, to try to reveal future directions for researches. Though there are a lot of other recent reviews on the topic [8,9,10], additional efforts were applied in this work to see the problem wider and possibly generate novel knowledge.

## 2. Organophosphorus Hydrolase

**Figure 2:**
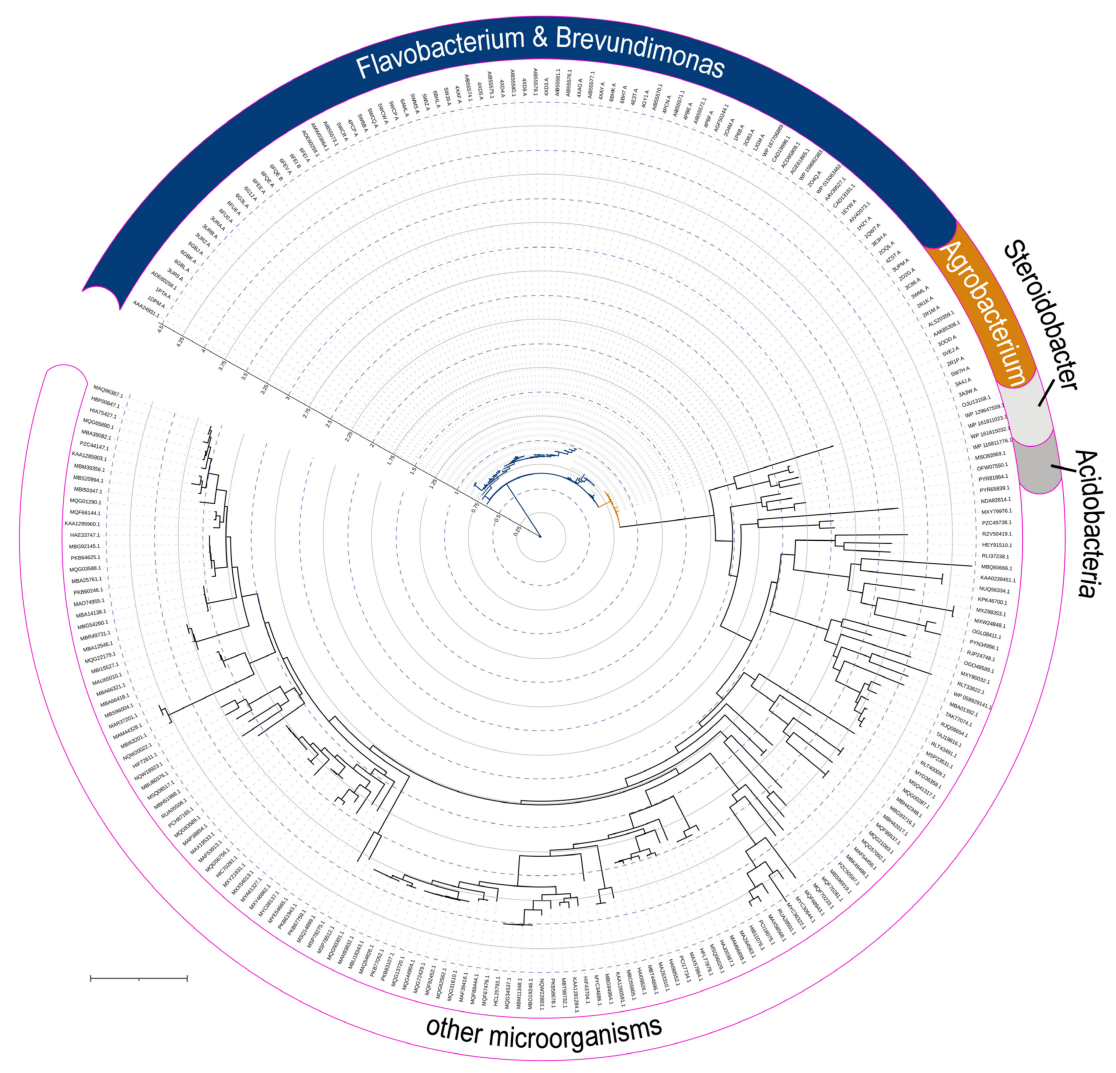
Phylogenetic tree of OPH and its homologous enzymes (down to 35% identity). Amino acid sequences were searched using BLAST (https://blast.ncbi.nlm.nih.gov/Blast.cgi) with PDB 1QW7 as a reference one; then they were multiply aligned with Clustal Ω (https://www.ebi.ac.uk/Tools/msa/clustalo/) and their tree was generated with IQ-TREE (http://iqtree.cibiv.univie.ac.at/); the tree was visualized with iTOL (https://itol.embl.de/). Though enzymes from *Flavobacteria*&*Brevundimonas* and *Agrobacteria* have similar high identity (89–100%), they were sub-divided and highlighted with blue and orange, respectively. Identifiers of individual transcripts/proteins according to NCBI/PDB are indicated on a roundabout. Internal scaling is shown for visualization purposes by solid grey and dashed blue circles.

Organophosphorus hydrolase (OPH; or phosphotriesterase, PTE) from the amidohydrolase superfamily is the most well-studied enzyme capable of hydrolyzing OPCs. OPH was firstly isolated from bacterial cells of *Pseudomonas diminuta* (currently classified as *Brevundimonas diminuta*) and *Flavobacterium* sp. which were extracted from a soil contaminated with parathion [1]. Hundreds of other enzymes having a high identity (i.e., the extent to which two amino acid sequences have the same residues at the same positions in an alignment and are expressed as a percentage) with OPH (Figure 2) were subsequently found in the genetic material of a wide variety of microorganisms, and are now grouped in a separate family of Phosphotriesterase-like lactonases (PLLs). Noteworthily, OPH is quite close to the root of the phylogenetic tree and, thus, can be evolutionary ancient.

**Scheme 1:**
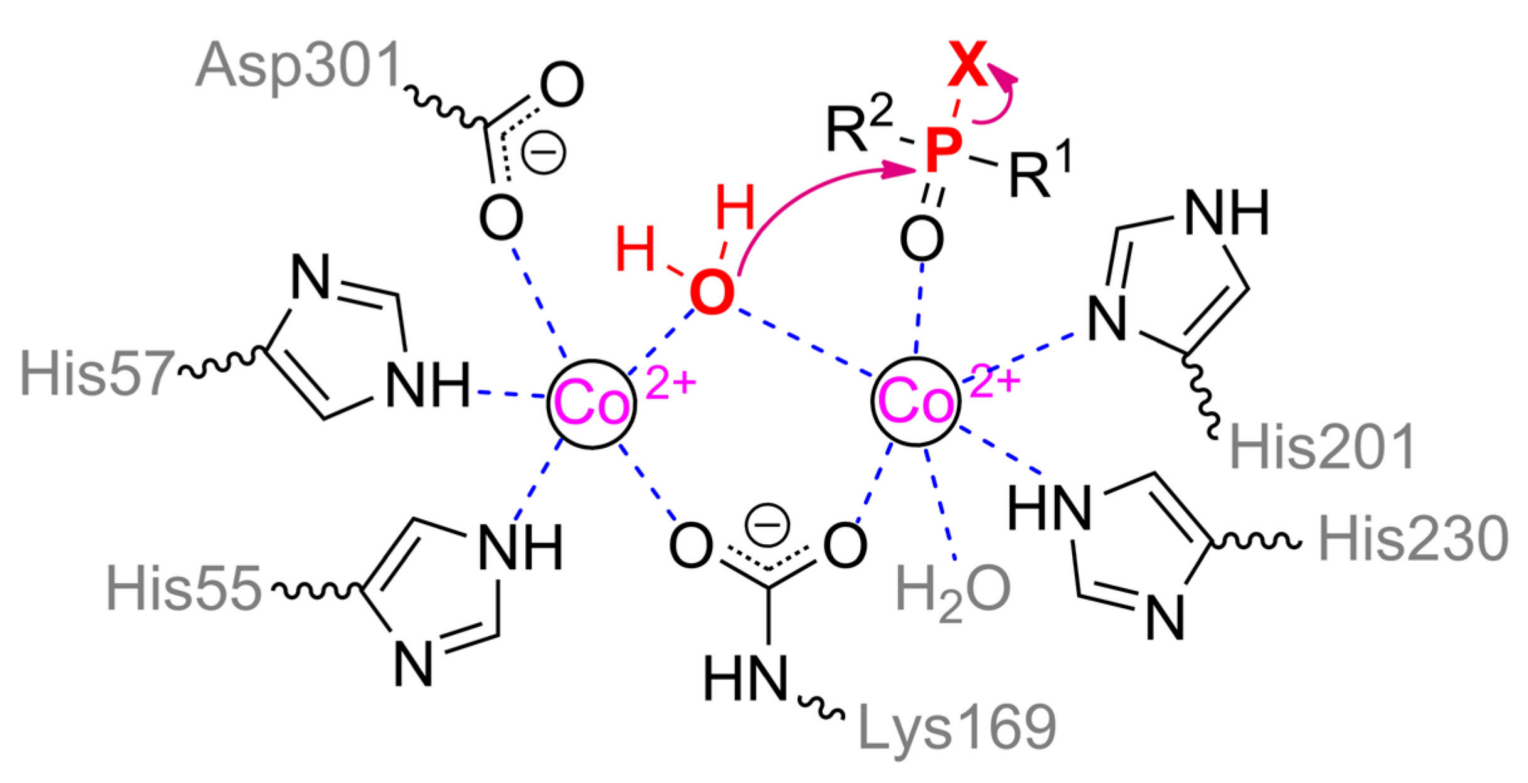
Mechanism of catalytic action of OPH.

Multiple crystallographic structures solved to date have a distorted (α/β)_8_ fold, i.e., TIM barrel (Figure 1a) [9]. Eight parallel β-strands form a core β-barrel and are alternated by eight α-helices with loops. The active center is assembled at the C-terminus of the β-barrel and contains two divalent metal ions. Zn^2+^ ions were found in the native OPH but the enzyme is fully active with Cd^2+^, Mn^2+^, and Ni^2+^ [1], and the maximal activity is observed with Co^2+^ [10]. The coordination sphere of the more buried α-metal ion includes two amino acid residues of histidine (His55 and His57 from β-strand 1) and aspartic acid (Asp301 from β-strand 8) and has a trigonal bipyramidal geometry (Scheme 1). β-metal ion is slightly more exposed to the solvent and surrounded by two histidines (His201 from β-sheet 5 and His230 from β-strand 6) which form an octahedral coordination sphere together with other amino acid residues and bound water molecules. The two metals are bridged by a water molecule (or hydroxyl-ion) and carboxylated lysine (Lys169 from β-strand 4), formed as a result of the interaction of Lys169 with CO_2_ dissolved in water [1].

Paraoxon is the best substrate for OPH, and the catalytic efficacy of its hydrolysis is close to the diffusion limit from the solvent, although the enzyme can also act on substrates with hydrolyzable P–O, P–F, P–S, and C–P bonds [11]. OPH prefers triesters as substrates which include phosphonate and phosphinate compounds in addition to phosphate esters [12,13] but has very low activity in reactions with di- and monoesters [12]. Moreover, even crystallographic structures with such substrates can be obtained [14].

Three hydrophobic pockets of the binding site are suitable to allocate three ester groups of the substrate. The pocket of leaving group consists of residues Trp131, Phe132, Phe306, and Tyr309, all of which pre-determine the enzymatic specificity for the leaving group [9]. Two other ester groups interact either with large pocket formed by His254, His257, Leu271, and Met317, or with small pocket formed by Gly60, Ile106, Leu303, and Ser308. These large and small pockets appeared to pre-determine enzyme specificity to the secondary ester groups of the substrate, including stereochemical preferences of substrates with chiral phosphorus centers, and, thus, enzyme stereoselectivity can be increased, decreased, or inverted by modifying these pockets [8].

*S*_P_-enantiomers of the most phosphate substrates are preferred by wild-type enzyme, and stereoselectivity increases with the size of these side groups. For example, when changing from isopropyl to cyclohexyl group, OPH prefers the *S*_P_-enantiomer over the *R*_P_-enantiomer in 130 and 78,000 times, respectively [8]. The same situation is observed with phosphonate compounds; with the only exception that *R*_P_-enantiomers become preferable. The selectivity of wild-type OPH to the *R*_P_-enantiomer when changing from isopropyl to cyclohexyl group is 20 and 760, respectively. The shrinking of small pocket by Gly60Ala mutation increases the enantiomeric selectivity for *R*_P_-enantiomer of cyclohexyl derivative from 760 to 23,000 times [15]. Double mutation His254Gln/His257Phe in large pocket reduces selectivity to 6. A simultaneous modification of both large and small pockets by mutation His254Gly/His257Trp/Leu303Thr inverts the selectivity so that the *S*_P_-enantiomer is 100 times more preferred. Such flexibility of OPH was proposed [8] to be useful for the isolation of enantiomerically pure compounds and for the selective degradation of enantiomers, though no practical examples have followed.

A rational design was implemented in a lot of studies to modulate enzyme activity towards certain substrates such as chemical warfare agents or pesticides [8]. Some success was achieved (e.g., activity was increased by more than 2 orders of magnitude with the most toxic enantiomers of sarin and soman, by about 25 times for VX, etc. [15]), but the ‘default values’ of kinetic parameters for paraoxon hydrolysis were never gained.

The combination of isotope-labeled substrates with structural, kinetic and physical research methods was used to study OPH mechanism of action. Using ^18^O-labeled water and substrates, it was demonstrated that the nucleophilic attack is targeted at the phosphorus center, and not at the leaving group [16]. The maximal primary isotope effect for *O*,*O*-diethyl ^18^*O*-(4-carbamoylphenyl) phosphate being equal to 1.039 and specified by a (de)protonation of the phenolic group in an aqueous medium, should correspond to the complete destruction of the chemical bond in the transition state. The value of 1.036 was experimentally observed and used as evidence that the bond with the leaving group is almost cleaved for this substrate already in a transition state. An even smaller primary isotope effect of 1.027 was observed via alkaline hydrolysis. The secondary isotope effects with a maximum value of 1.04 could be determined when bond order between oxygen and phosphorus (P=^18^O) changes from 2 to 1. An alkaline hydrolysis of the substrate labeled at phosphoryl oxygen led to the secondary isotope effect of 1.025 which is equal to a bond order of ca. 1.4 in a transition state. The secondary isotope effect for the enzymatic reaction decreases to 1.0181 which is equal to a bond order of 1.53 and attributed to the coordination with metal ion [16].

Interestingly, the primary and secondary isotope effects for the best substrate, i.e., paraoxon, are much more modest during chemical hydrolysis and practically disappear during enzymatic action. Thereby, this confirms the fact that neither the productive substrate binding with the active center, nor the breakaway of the leaving group, are speed-limiting stages of the enzymatic process.

The presence of a water or hydroxyl-anionic bridge between metals is clearly visible on the X-ray structure of the OPH active center (PDB 1QW7). The probing of the binuclear active site containing Mn^2+^ by X-band EPR spectroscopy has demonstrated the hyperfine splitting of the Mn^2+^ signal [17]. When binding of *O*,*O*-diisopropylmethylphosphonate or (to a lesser degree) triethyl phosphate but not diethyl phosphate, there is a significant change of hyperfine structure near *g* = 2.2 and an appearance of hyperfine splitting near *g* = 3.8 and 4.3. Though disturbance by diethyl phosphate is much more modest, this OPC also somehow changes EPR spectra near *g* = 2.2 and 3.8. Currently, a hyperfine structure spaced at 40–45 G intervals near *g* = 2.2 is considered to be characteristic for enzymes containing binuclear spin-coupled Mn^2+^ ions. Moreover, the main peak at *g* = 2 is highly likely to be a signal from quintet state (*S* = 2) transitions [18], while peak at *g* = 4 can be superimposition of *S* = 1 and *S* = 2. Though authors [17] mainly speculated about some ‘lowered symmetry’ and ‘mononuclear Mn(II) signal’ and attributed above-mentioned *g* values to these processes, someone could agree that coordination spheres of both metals have certainly changed during substrate binding. Thus, coordination of phosphoryl oxygen and β-metal dramatically affects its environment and even after displacement of bridge water / hydroxyl-ion it will not disturb metal coupling since there is a carboxylated lysine bridge, preventing metal uncoupling. Moreover, exactly lowering a pH to ca. 6 would decarboxylate this residue [19] and disturb a geometry of metal coordination that could lead to observed EPR signal decoupling [17].

A catalytic p*K*_a_ ~ 6 determined from the OPH kinetic pH profile has been attributed for a long time [8] to correspond to a p*K*_a_ for protonation of the bridging hydroxyl group for the reaction to occur. Alternatively, this value may be due to (de)protonation of the His254 residue, capable of interacting with Asp301 and pulling its proton appearing along with substrate catalytic transformation. Particularly, the study of a close homolog of OPH from soil bacteria *Agrobacterium radiobacter* (OpdA), which has more than 90% amino acid sequence identity and completely identical structure of the active center (Figure 1b), using magnetic circular dichroism has showed [20] that the water molecule bound to the α-metal in the Co^2+^-containing enzyme can serve as an attacking nucleophile instead of a hydroxyl group at elevated pH values. Nevertheless, such an attack is unlikely to occur in the presence of Zn^2+^ and/or Cd^2+^ ions in the active center [21]. Moreover, the coordination of substrate analogs by one or both metals strongly depends on the structure of this compound [22], and therefore, these crystallographic data should be used carefully.

The p*K*_a_ of the leaving group in the phosphate or thiophosphate substrate should be less than 7.5 for the most effective catalysis (i.e., this group should be maximally charged under the reaction conditions for it to be successfully broken away from the phosphorus center) [23]. A break of the linear Brønsted dependence (catalytic activity vs. p*K*_a_) and a sharp decrease in the catalytic constant, as well as deterioration of the Michaelis constant, are observed at p*K*_a_> 7.5. These trends are altered insignificantly when metal ions in the active center are replaced but are generally preserved. Interestingly, such bends are not observed for phosphothiolate substrates (e.g., malathion and VX). From a practical standpoint, the effective p*K*_a_ of the leaving group can be modified by addition of supplementary agents into the medium, for example, nitrogen-containing compounds having their own p*K*_a_> 9 [24].

Recently, computer simulations [25] have become an integral part in the study of the mechanisms of enzyme action, and the entire dimer molecule or only part of it can be involved along this course. Multiple works were realized with such modeling of OPH. Thus, a system of two transition states during the hydrolysis of methyl paraoxon has been revealed by quantum-mechanical calculations using density functional theory (DFT) method applied only to a bridging hydroxyl ion, metals, and ligands emulating amino acid residues coordinating the metals [26]. When additional potentials emulating the dielectric constant of the medium (up to the water level) are applied, an additional intermediate in which the phosphorus surroundings have a trigonal bipyramidal geometry appears between transition states. According to the simulation, the first transition state is a bound substrate with a distorted phosphorus center, which allows bringing the phosphorus center and the hydroxyl group in a close proximity followed by the formation of a bond. Next, the hydroxyl proton is pulled to Asp301, the bipyramid is ultimately deformed into a distorted tetrahedron, and the bond with the leaving group is broken. The highest energy barrier is observed for the first transition state and reaches 10.5 kcal/mol, which together with the second barrier (2.2 kcal/mol) gives a comparable value with a 12.8 kcal/mol determined experimentally [27]. Interestingly, a similar energy barrier of ca. 11 kcal/mol was observed for the protonation of hydroxyl ion bridging two Mg^2+^ ions [28], followed by uncoupling of metals with this water molecule.

The entire enzyme dimer, including water molecules in its active center and in the solvate shell, can be included in the analysis during the combined quantum-mechanical / molecular-mechanical (QM/MM) modeling [27,29]. In toto, the results of the calculations [27] were similar to the previous ones obtained only with DFT: the high-energy first transition state formed as a result of the nucleophilic attack of hydroxyl/water on the phosphorus center, transforms into an enzyme-product complex with a small barrier, and subsequent dissociation occurred. Although the calculated total energy barrier of 18.3–21.4 kcal/mol was significantly higher than the experimentally determined one, this simulation has predicted that the bridging hydroxyl group is firstly disengaged from the β-metal, then from the α-metal, and then leaves any possible interaction with Asp301, while forming a bond with phosphorus in the transition state. At the same time, the distance between metal ions increases that, apparently, contributes to the correct orientation of the phosphorus center for nucleophilic attack. At the end of the transformations, the water molecule diffuses from the surrounding solvent into the active center and becomes a bridging group, pushing the product out of the active site.

Interestingly, the His254 residue is deprotonated according to this calculation when a leaving group goes out from the active center. His254 is believed to transfer a proton to Asp233 from Asp301 (the total distance is more than 6 Å), which, in turn, modulates the formation of a hydroxyl bridging group from a water molecule. At the same time, there may be steric hindrances for such a proton transfer mechanism due to the fact that it is oriented in the opposite direction from Asp233 [27]. Therefore, His254 is more likely to be a direct source for protonation of the leaving group. In particular, the amino acid mutations His254Asn and especially His254Ala [30] have resulted in a significant (3–6 times) increase in the catalytic efficiency with *O*,*O*-diethyl *O*-(4-chlorophenyl) phosphate having a poor leaving group (p*K*_a_ = 9.38), which should be maximally deprotonated for the best catalysis. The opposite situation is observed in the case of paraoxon having a strongly charged leaving group (p*K*_a_ = 7.14): the regeneration of the attacking nucleophile (hydroxyl group) in the active center becomes a rate-limiting process, and the enzyme efficiency deteriorates by 4–8 times with the same replacements.

Though a more detailed study by the same QM/MM method has revealed a number of additional intermediate states during substrate transformation [29], their general sequence remained the same. Moreover, the maximal energy barrier of 18.7–21 kcal/mol is also upon nucleophilic attack and is comparable with the previous study.

Interestingly, the authors of both studies [27,29] have limited themselves to only a small part of the enzyme molecule with a radius of no more than 25–30 Å from the phosphorus center. At the same time, OPH homodimer (approximately ellipsoidal in shape with a radius of 30 Å and a length of 100 Å) existing under real conditions in solution is dynamically and conformationally more mobile [31]. The shift from the dimeric to monomeric form can affect the binding of substrates and, accordingly, the specificity of the enzyme, and, therefore, this should be taken into account during further similar studies using computer simulation.

## 3. Methyl Parathion Hydrolase

**Figure 3:**
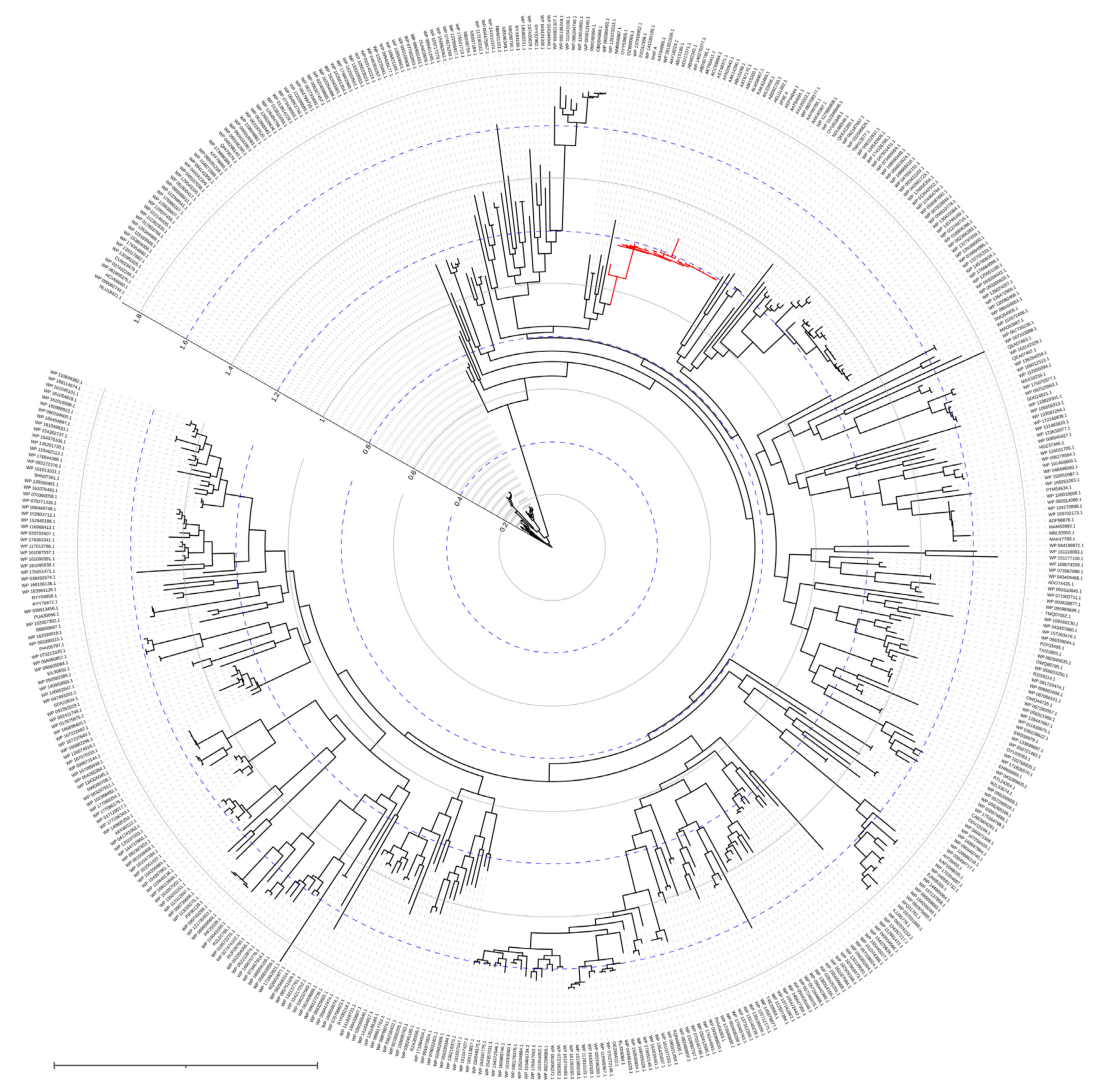
Phylogenetic tree of MPH and its homologous enzymes (down to 50% identity). The tree was built as previously with PDB 1P9E as a reference sequence. Enzymes having high identity (89–100%) are highlighted with red.

Methyl parathion hydrolase (MPH) was isolated from several soil bacteria of the genus *Pseudomonas*, which can use methyl parathion as the only source of carbon [32]. Though the bacteria were isolated from different soils and sites, the sequences of the MPH variants have surprisingly high identities (over 98%). Although these enzymes were originally thought to be homologous to OPH but their amino acid sequences appeared to have only 43% homology and 33% identity. Typical MPH from *Pseudomonas* sp. WBC3 is a homodimer [33], and each monomer has an αβ/βα sandwich fold (Figure 1c) being typical of the metallo-hydrolase/oxidoreductase superfamily which also includes β-lactamases and acyl homoserine lactonases. Phylogenetic tree reconstruction has placed MPH in the middle of this wide superfamily (Figure 3).

Nevertheless, the active site of MPH has common features with OPH, including the presence of three hydrophobic domains to accommodate substrate side-groups, though there are differences also. The leaving group can interact with amino acid residues Phe119 and Phe196 [32], while Trp179 is likely to be used for proper orientation of Phe196 and/or other residues. As a result, replacement of any of them with alanine led to a deterioration of both catalytic constant and Michaelis constant (excepting the case with Phe119Ala where *K*_m_ is almost retained). The large pocket is formed by Pro68, Val69, Arg72, Leu258, and Leu273, and the small one is formed by Leu65, Leu67, and Val97.

α-Metal is coordinated by His152, His302, and Asp151 (Figure 1c) and coupled with β-metal via a carboxylate ligand (Asp255) and a bridging water (or a hydroxyl anion). β-metal has an octahedral coordination sphere formed by His147, His149, and His234 together with a water molecule and two bridging ligands. The carboxylate bridge binds the α- and β-metals through a single oxygen atom, and thus a hypothesis that the other carboxylate oxygen can potentially act as an additional ligand to the α-metal, has been proposed [8]. However, in the case of such ‘double’ coordination, the coordination sphere of the α-metal will be a distorted pentagonal pyramid, which is cardinally different from those being characteristic for Zn^2+^ in active centers of hydrolases [34]. Therefore, a trigonal bipyramid with the only oxygen of Asp255 seems to be more likely.

The vigorous similarity between active sites of enzymes and the environment of metals, including the presence of bridging water, suggests that the catalytic mechanism of MPH is similar to that known for OPH [32] (Scheme 1).

## 4. Other Enzymes of PLL Family

**Figure 4:**
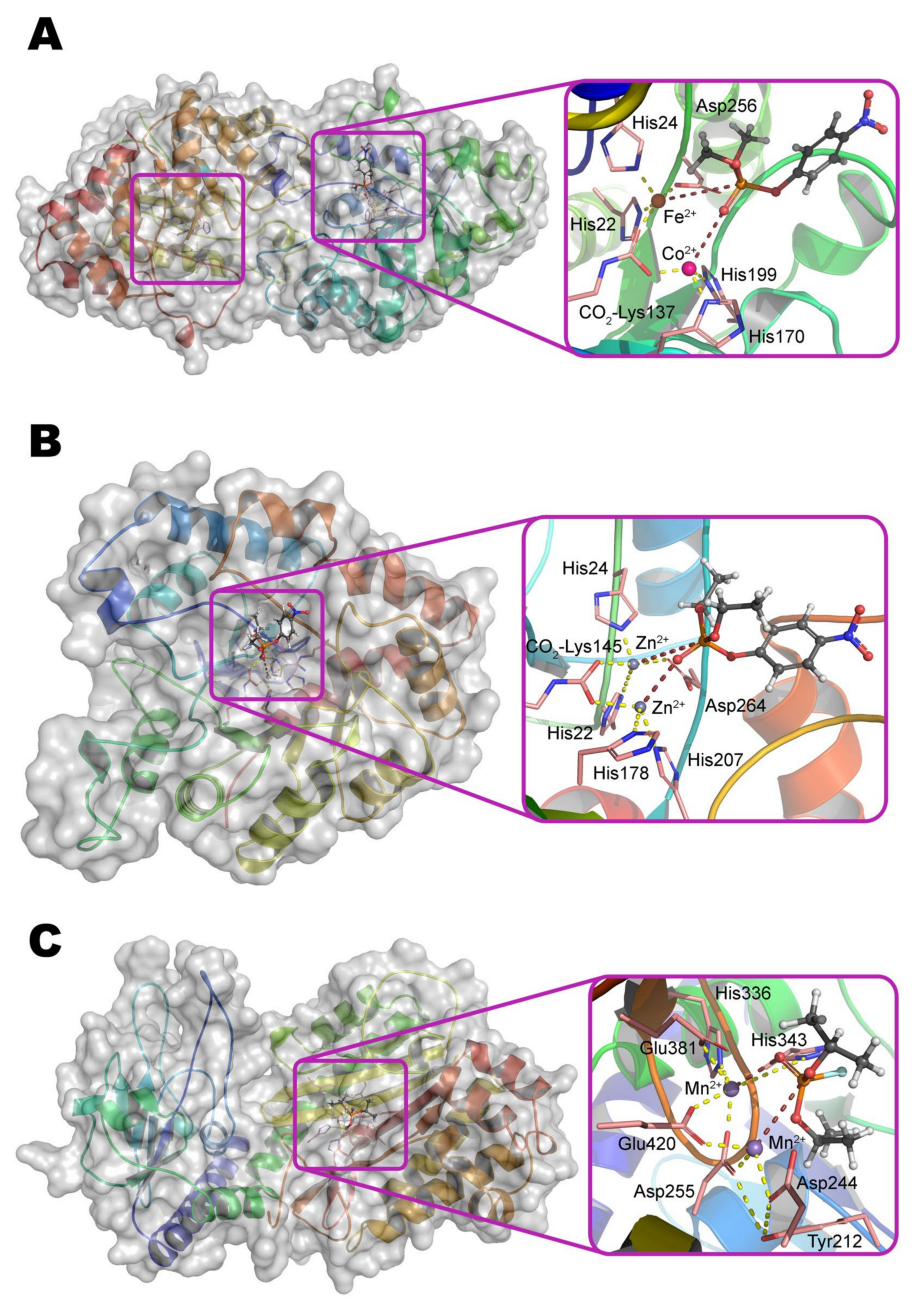
The structure of SsoPox dimer (**A**), mPHP monomer (**B**), and OPAA dimer (**C**) according to X-ray diffraction data (PDB 3UF9, 4IF2, and 3L24, respectively). The entrance to the active centers of the enzymes is framed, and their structures are shown in detail within insets. The binding of the substrate methyl paraoxon (**A**), paraoxon (**B**), and diisopropyl fluorophosphate (**C**) was determined as previously. The coordination bonds of metal ions with amino acid residues in active centers, as well as with labile groups of the substrate are shown by a dashed line.

The crystal structures of most lactonases of the PLL family have not yet been determined, and many of these enzymes have not even been biochemically characterized. However, those enzymes which have been studied well enough [9], have a similar active center as compared with OPH. Perhaps, the most studied and interesting lactonases are SsoPox from archaea *Sulfolobus solfataricus* [35] and mPHP from bacteria *Mycobacterium tuberculosis* [36] (Figure 4a,b).

As compared with OPH, SsoPox (49% homology, 31% identity) appears to be slightly more elongated in the direction of the largest axis, but the TIM barrel folding is preserved. Though the active site of these enzymes is formed by the same amino acid residues, SsoPox contains Fe^2+^ and Co^2+^ ions within it (Figure 4a). The catalytic constant of SsoPox appeared to be 2 orders of magnitude worse than in the case of OPH, while Michaelis constant towards best substrate (paraoxon) degraded in 3 orders of magnitude (possibly due to the ‘flattened’ active center). Altogether it leads to a catastrophic decrease of SsoPox activity in comparison with OPH (more than 3 orders of magnitude). Moreover, SsoPox activity in reactions with substrates containing P=S group (e.g., methyl parathion, chlorpyrifos, etc.) decreases much faster than in the case of OPH. However, SsoPox exhibits an outstanding thermal stability as well as persistence against the action of various denaturing agents (detergents, chaotropes, organic solvents). This may be due to both a solid folding and additional optimization(s) of the amino acid sequence. For example, the Ala259–Arg275 loop of OPH was reduced to the stiffer and shorter Leu228–Pro231 in SsoPox. At the same time, some of the amino acid residues of this loop in OPH are involved in the formation of a large pocket for substrate binding.

Besides, numerous salt bridges on SsoPox surface can contribute somehow [37]. Interestingly, amino acid residues Arg223 and Leu226 in SsoPox correspond to His254 and His257 in OPH, respectively. While double substitution His254Arg/His257Leu in OPH is known from the literature [38] to lead to an 11-fold decrease in the catalytic constant for paraoxon. Some improvement of the Michaelis constant cannot compensate for such a strong decrease in the catalytic constant, and as a result, the efficiency constant decreases 8-fold. At the same time, the catalytic constant for V-gases and their analogs increase by 10–39 times with this substitution.

Thus, native SsoPox is initially more adapted to the hydrolysis of substrates with a long and/or branched leaving group, for example, those that are present in the structure of its natural substrates (*N*-acyl homoserine lactones, γ- and δ-lactones, etc.) [39].

The Ala259–Arg275 loop of OPH is reduced to Val236–Pro239 in mPHP (51% homology and 34% identity to OPH) also. However, an extended His108–Glu120 domain appears additionally in place of Arg139–Val143 of OPH (Figure 4b). This site is located at the junction of two subunits in a homodimer, and therefore the stability of such protein molecule can be further increased. This enzyme also contains His254Arg/His257Val substitutions (according to OPH numbering), and the activity of mPHP containing Zn^2+^ ions is reduced by another 2 orders of magnitude towards paraoxon as compared to SsoPox [36].

It is assumed that catalytic mechanism of both SsoPox and mPHP as well as other lactonases of the PLL family is similar to the previously considered mechanism of OPH (Scheme 1) due to the similarity in the structure of the active centers.

**Figure 5:**
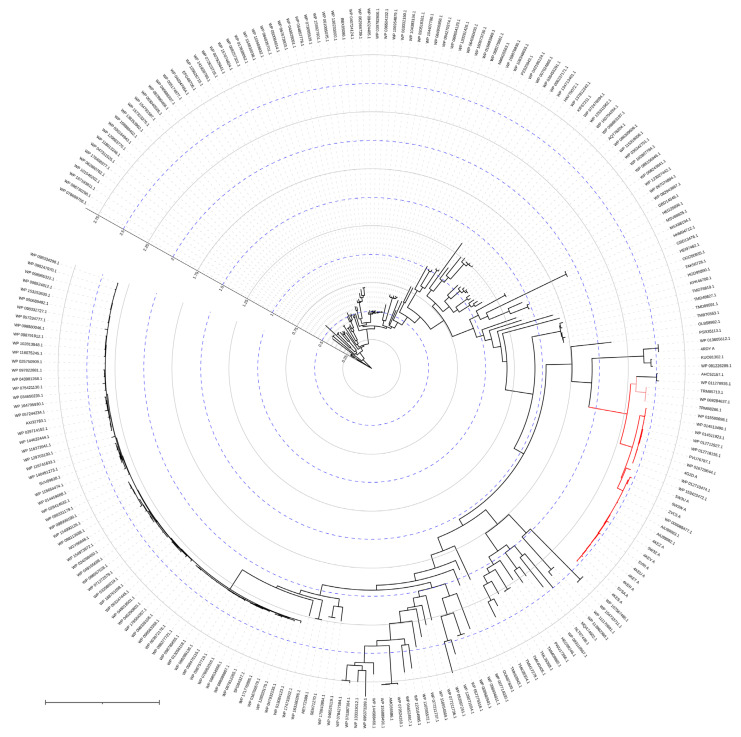
Phylogenetic tree of SsoPox and its homologous enzymes (down to 40% identity). The tree was built as previously with PDB 3UF9 as a reference sequence. Enzymes having high (90–100%) and good (80–89%) identity are highlighted with red and pink, respectively.

**Figure 6:**
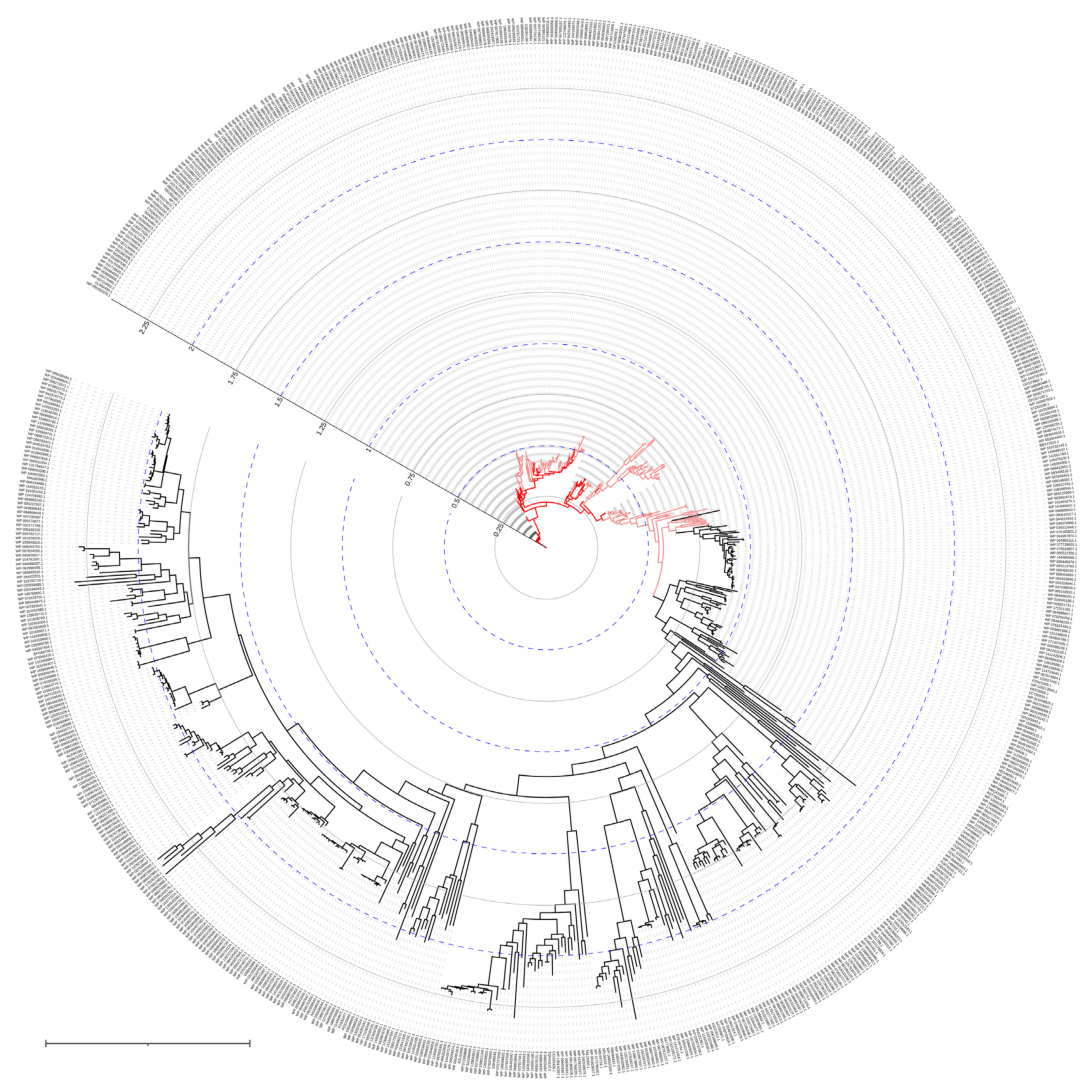
Phylogenetic tree of mPHP and its homologous enzymes (down to 55% identity). The tree was built as previously with PDB 4IF2 as a reference sequence. Enzymes having high (90–100%) and good (80–89%) identity are highlighted with red and pink, respectively.

Interestingly, SsoPox forms a narrow cluster in the phylogenetic tree (Figure 5), while mPHP is a basal variant (Figure 6) as in the case of OPH. That emphasizes evolutional significance of both OPH and mPHP for microorganisms.

## 5. Organophosphorus Acid Anhydrolase

**Scheme 2:**
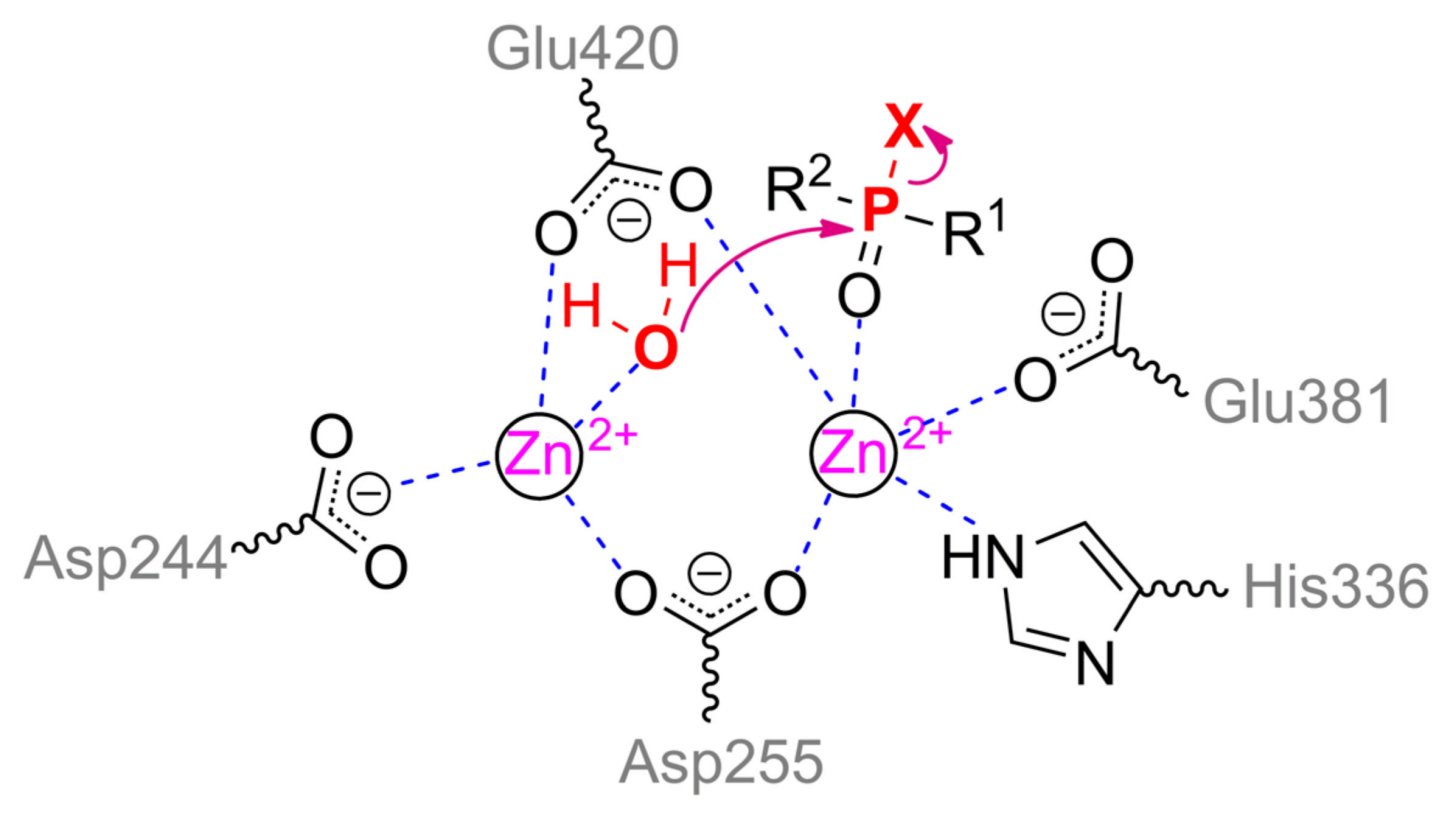
Mechanism of catalytic action of OPAA.

Another enzyme being active towards OPCs is organophosphorus acid anhydrolase (OPAA) from bacteria *Alteromonas* sp. [1]. OPAA belongs to prolidases (a superfamily of aminopeptidases). PepQ [40] and aminopeptidase P (AMPP) from bacteria *E. coli*, which also has a small OPH-activity [41], are also prolidases. Though the homology / identity between AMPP (or PepQ) and OPAA are 42% / 31% (or 67% / 50%), respectively, the structure of their active centers is completely the same. OPAA forms a homodimer, which combines with analogous dimer to form a tetramer [42]. The pita-type fold is formed from the C-terminus of the protein globule, and a binding site for two metal ions is located in its central cavity (Figure 4c). The maximum activity is observed with Mn^2+^, which, however, does not exclude the possibility of other bivalent metals excepting Cu^2+^ and Ca^2+^ [43]. Interestingly, the thermostable homolog from archaeon *Pyrococcus horikoshii* [44] shows maximal activity with Co^2+^ and not Mn^2+^. The α-metal is slightly more exposed to the solvent and coordinated by His336 and Glu381 residues, while the β-metal is coordinated by Asp244 and attacking nucleophile (Scheme 2). Both metals are coupled by bridging Glu420 and Asp255, which, thereby, complete the coordination sphere of the α- and β-metal to the trigonal bipyramid and tetrahedron, respectively. His343 residue is located similarly to His254 in OPH and, therefore, can perform similar functions of protonation of the leaving group. Solved OPAA structure with a bound product showed a coordination bond between the α-metal and phosphoryl oxygen. It is believed that while a substrate or product is absent, metals are also connected by a bridging water molecule found in the AMPP structure [45].

**Figure 7:**
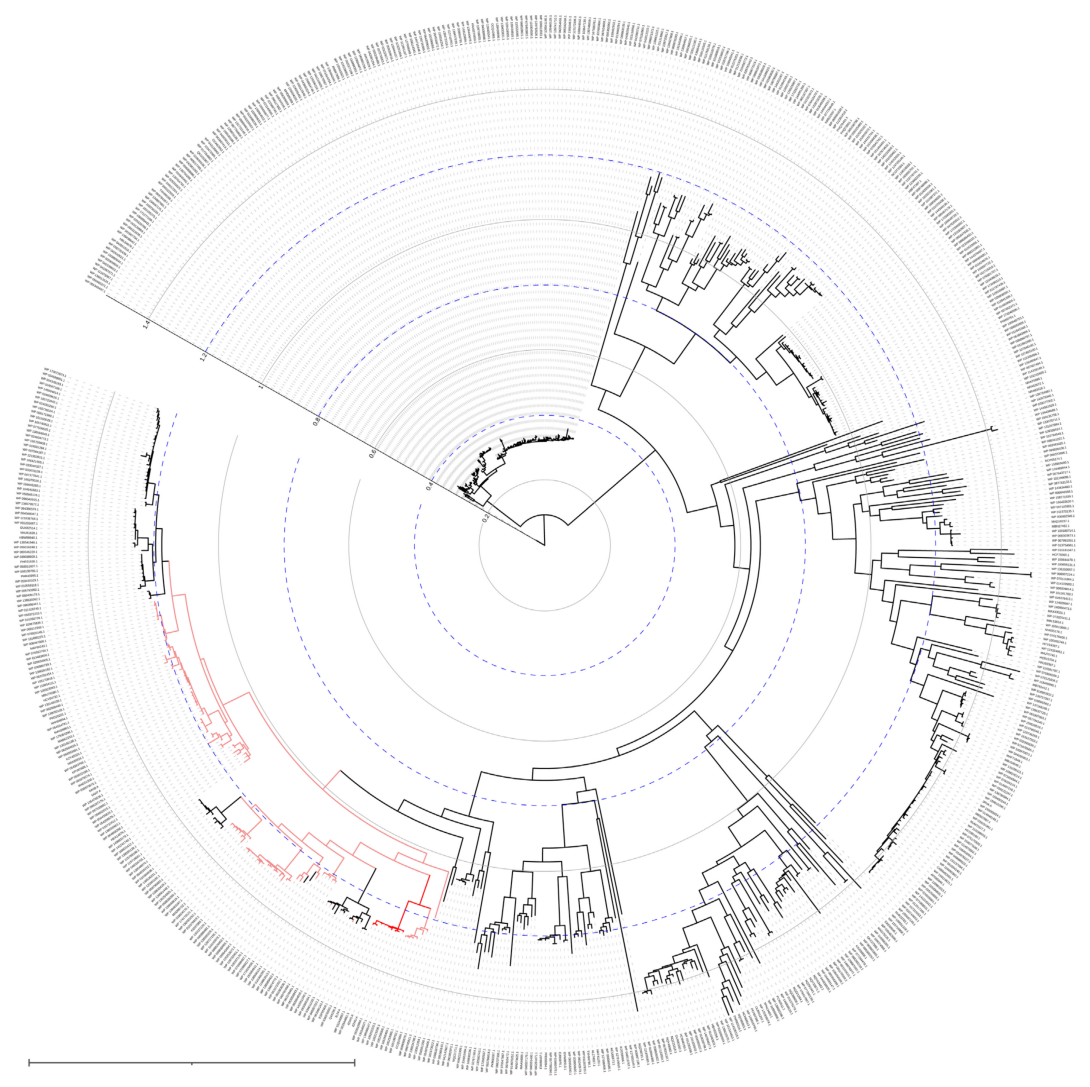
Phylogenetic tree of OPAA and its homologous enzymes (down to 56% identity). The tree was built as previously with PDB 3L24 as a reference sequence. Enzymes having high (90–100%) and good (80–89%) identity are highlighted with red and pink, respectively.

Being prolidases, both OPAA and AMPP have native activity towards proline-containing peptides produced by the cleavage of collagen-like proteins. However, OPAA is actively evolving (Figure 7). This could explain why OPAA activity with fluorine-containing phosphotriesters is five times higher than with dipeptide substrates, and the maximal catalytic activity is observed with soman. OPAA also catalyzes the hydrolysis of other OPCs, for example, those containing a *p*-nitrophenol leaving group, though in orders of magnitude slower than OPH. Native OPAA has the highest stereoselectivity towards *R*_P_-enantiomers of methylphosphonates, which can reach values of more than 7000 in the case of soman analogs [42]. Interestingly, the stereoselectivity of AMPP is reversed, and *S*_P_-enantiomers of methylphosphonates are preferred [41].

OPAA substrate-binding site consists of three pockets as in the case of OPH: a small pocket, a large pocket, and a leaving group pocket. The small pocket is formed by residues Tyr212, Val342, His343 from one subunit and Asp45 from the second subunit. Tyr212 can also form hydrogen bonds with catalytically significant Asp244 and Asp255. In particular, the substitution Tyr212Phe increases the efficiency of the enzyme towards Russian VX (VR) by five times [46]. The large pocket is inlayed with residues Leu225, His226, His332, Arg418 from one subunit and Trp89 from the second subunit. Besides, Arg418 forms a hydrogen bond with Asp244 and plays a significant role in the correct assembly of the active site: the replacement of Arg418Ala leads to a complete loss of catalytic activity [43]. With the exception of His226 and His332, the replacement of any of above-mentioned residues by alanine decreases the enzyme activity with OPCs by multiple times. The activity remains approximately at the initial level in the case of substitutions His226Ala and His332Ala [43]. Though double replacement Asp45Trp/His226Gly results in 30-fold increase of *k*_cat_ towards paraoxon while having a negligible effect on *K*_m_ [47]. Interestingly, such modification decreases the native peptidase activity nearly 4-fold. According to QM/MM simulations, uncoupling of Arg367 with both Asp45 and His226 could be the reason and, thus, this residue can interact with the leaving group and orient it properly.

In general, the mechanism of both OPAA and AMPP is similar to that of OPH: the substrate is bound through phosphoryl oxygen to the α-metal, followed by some shift of the nucleophile, deformation of the microenvironment of the phosphorus center, and a nucleophilic attack on it (Figure 4c).

## 6. Paraoxonase

**Figure 8:**
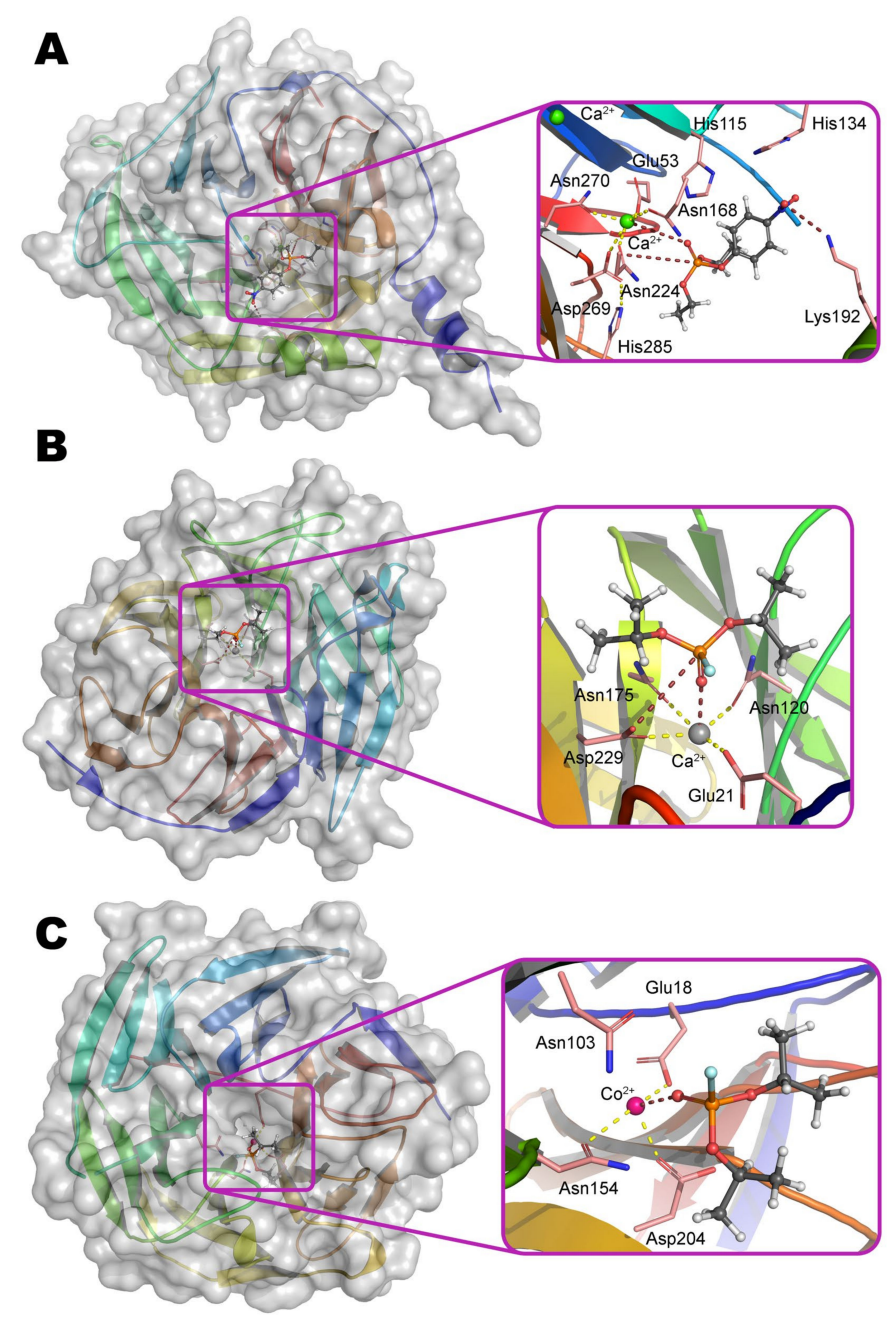
The structure of modified PON1 (**A**), DFPase (**B**), and SMP30 (**C**) according to X-ray diffraction data (PDB 1V04, 2GVV, and 3G4E, respectively). The entrance to the active centers of the enzymes is framed, and their structures are shown in detail within insets. The binding of the substrate paraoxon (**A**) and diisopropyl fluorophosphate (**B**,**C**) was determined as previously. The coordination bonds of metal ions with amino acid residues in active centers, as well as with labile groups of the substrate are shown by a dashed line.

Serum paraoxonase (PON) of mammals, as the name suggests, is present in the serum as well as in other organs and tissues of mammals. This enzyme in humans has three isoforms differing in their specificity to different substrates, in their distribution in the body, in the level of expression, etc. [48]. PON1 (Figure 8a) among these three isoforms has the highest activity towards OPCs. Though the OPH-activity of PON1 is an order of magnitude lower as compared to its arylesterase (lactonase) activity, a large amount of research of this enzyme was carried out exactly with OPCs.

**Figure 9:**
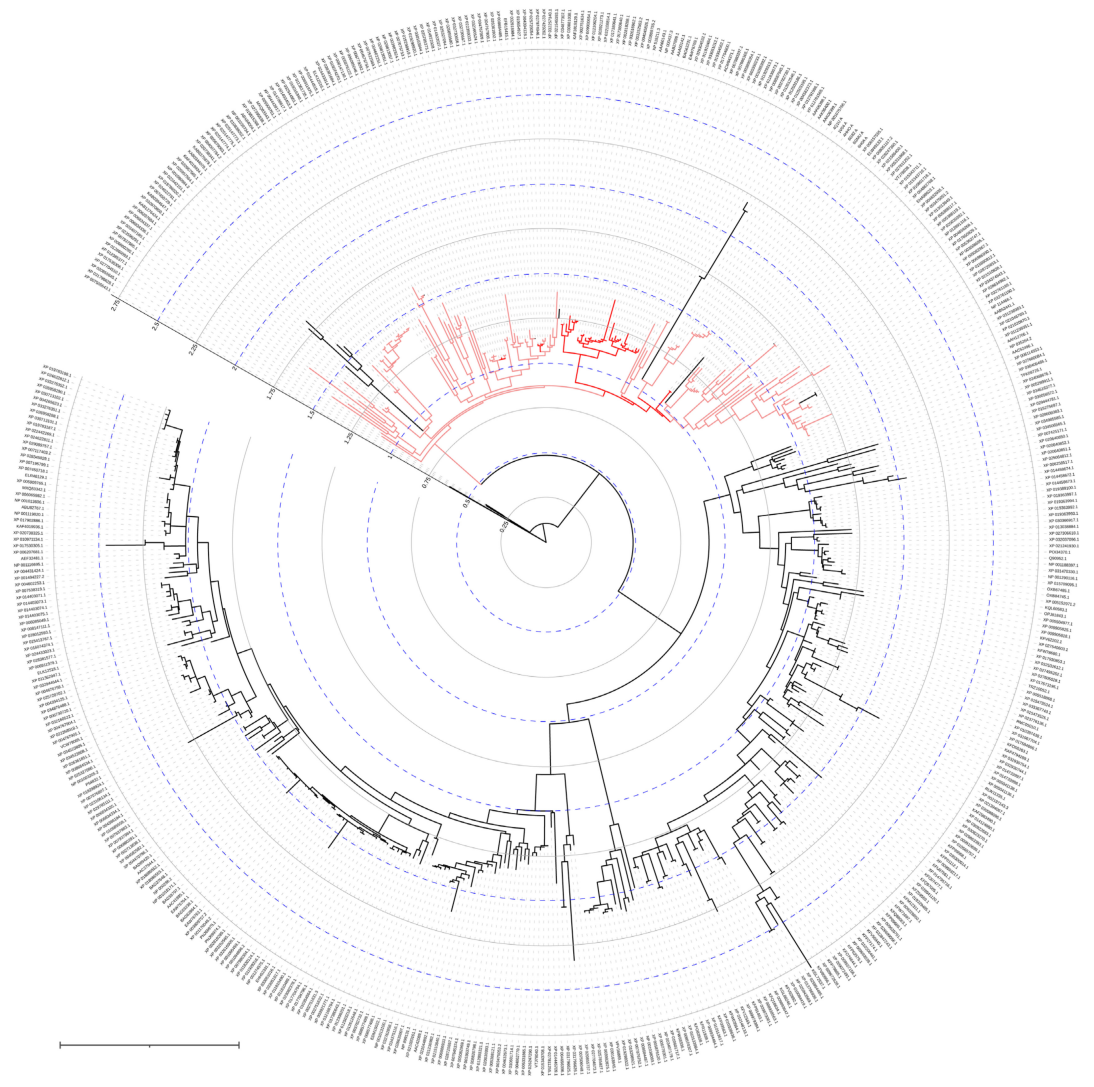
Phylogenetic tree of hPON1 and its homologous enzymes (down to 59% identity). The tree was built as previously with PDB 1V04 as a reference sequence. Enzymes having high (90–100%) and good (80–89%) identity are highlighted with red and pink, respectively.

Interestingly, human PON1 is quite similar to the PONs of other animals (Figure 9) with very few exceptions, for example: clusters of marine mammals (fin whale, baiji, Yangtze finless porpoise, bottlenose dolphin, beluga whale), American pikas and Philippine tarsier.

PON1 belongs to the six-bladed β-propeller superfamily with appropriate folding and contains two Ca^2+^ ions (Figure 8a). OPH-activity of PON1 is greatly reduced in the presence of other bivalent metals (Ba^2+^, Cu^2+^, Mg^2+^, Sr^2+^) [49]. Each propeller blade is formed from four-stranded β-sheets, and two Ca^2+^ ions are located in the central cavity. It is assumed that the slightly more buried ion performs a structural function and is necessary for the correct folding of the protein, but is not needed for the catalysis directly. It is coordinated by residues Asp54 and Asp169, as well as three water molecules.

**Scheme 3:**
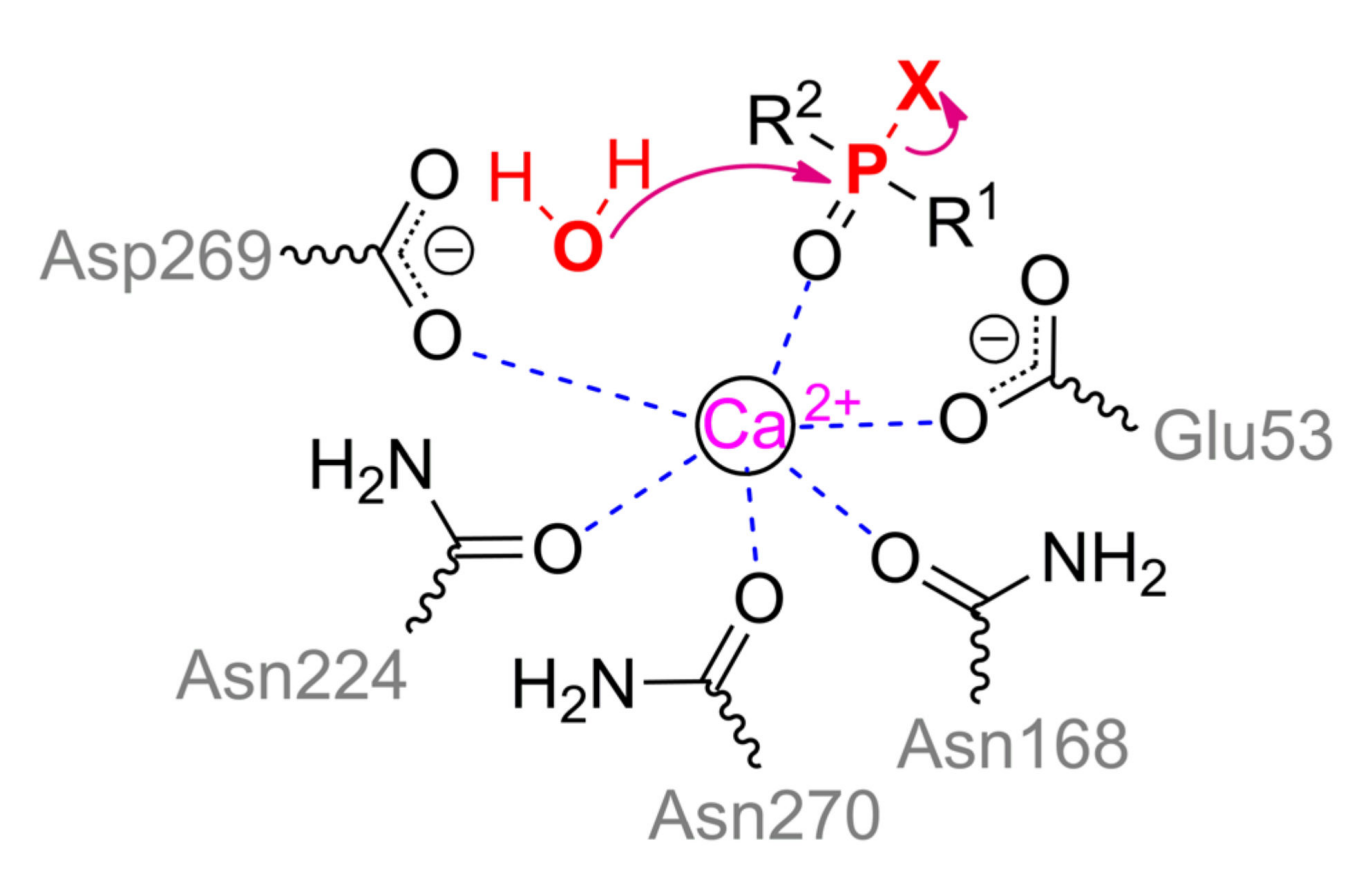
Mechanism of catalytic action of PON1.

Ca^2+^ ion being slightly more exposed to the solvent actually performs the catalytic function. It is coordinated by Asp269, Glu53, and three asparagine residues (Asn168, Asn224, and Asn270) (Scheme 3). The substrate bound through phosphoryl oxygen brings the coordination sphere of the metal to octahedral. His285 is capable of pulling a proton away from Asp269, which, in turn, activates a water molecule for a nucleophilic attack on the phosphorus center, or makes such an attack itself.

On the other hand, His115 and His134 were shown to be absolutely necessary for lactonase activity, and a single (His115Trp) or double substitution (e.g., His115Trp/Arg192Lys) multiplies the OPH-activity of PON1 [50]. It should be noted here that there is a single nucleotide polymorphism Gln192Arg in the human population which greatly improves catalytic activity of PON1 towards some OPCs and other substrates [51].

The pH-profile indicates that one group should be deprotonated with p*K*_a_ ~ 7. Thus, such deprotonated residue can be any of these histidines, though molecular dynamics modeling of the PON1 complex with lipids within high-density lipoproteins recognizes the greatest contribution of His285 to the OPH-activity of this enzyme [52].

According to QM simulations [53], the single transition state with trigonal bipyramidal geometry of the phosphorous center was observed. The energy barrier in the reaction course have varied greatly depending on substrate, and it was minimal with paraoxon (0.8 kcal/mol) and maximal with DFP (7.9 kcal/mol). Moreover, the reaction was slightly thermodynamically unfavorable in the case of DFP. Overall these results were in agreement with enzyme catalytic activity and preference to paraoxon.

## 7. Diisopropyl-Fluorophosphatase

**Figure 10:**
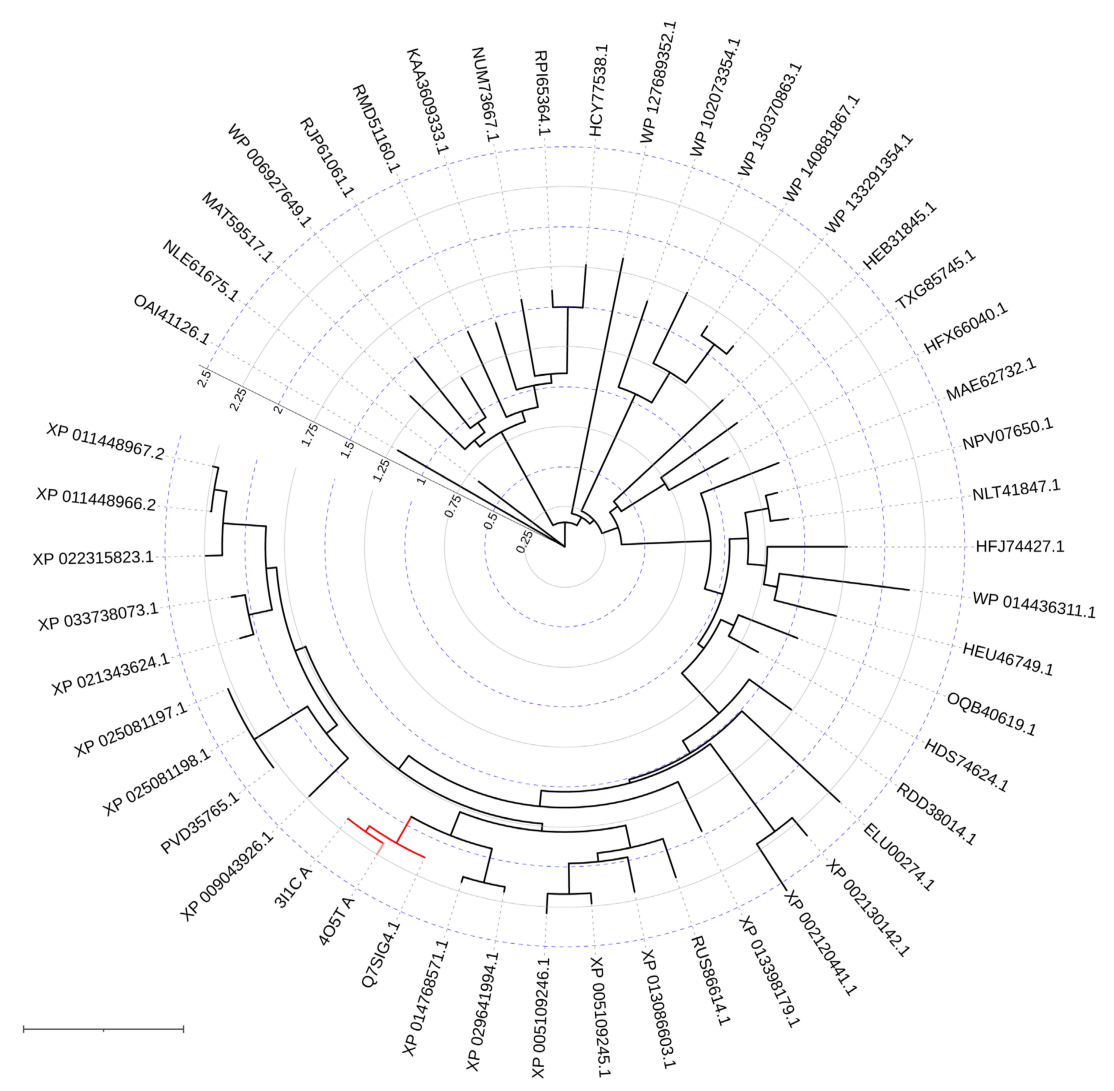
Phylogenetic tree of DFPase and its homologous enzymes (down to 30% identity). The tree was built as previously with PDB 2GVV as a reference sequence. Enzymes having high (90–100%) and good (80–89%) identity are highlighted with red and pink, respectively.

Diisopropyl-fluorophosphatase (DFPase) from squid *Loligo vulgaris*, in spite of it has very low homology (~ 10%) of the amino acid sequence with PON1, is structured similarly (Figure 8b). Two Ca^2+^ ions are also located in the central part of the six-bladed β-propeller fold. Structural Ca^2+^ is coordinated by Asp232, His274, and three water molecules; carbonyl oxygen of Leu273 completes the octahedral coordination sphere. The catalytic Ca^2+^ is coordinated by residues Glu21, Asp229, Asn120, Asn175, and two water molecules. Together with the substrate bound through phosphoryl oxygen, the coordination number becomes quite unusual and equal to 7 (a prism, one base of which is a square, and the other is an equilateral triangle). As in the case of PON1 (Scheme 3), the Asp229 residue activates a water molecule for nucleophilic attack or carries it out itself [54]. Proton can migrate along the Asp229–Ser271–His287 chain, and all these residues are catalytically significant, according to the study of mutant forms of DFPase. An interesting difference between DFPase and PON1 is the absence of an α-helix at the N-terminus, which is necessary for PON1 to be integrated into the lipid layer of high-density lipoproteins. Different metabolic pathways in mollusks (Figure 10) could contribute also.

All these modifications lead to a shift in substrate specificity, and DFPase catalyzes the hydrolysis of P–F (in DFP, sarin, soman) and P–CN (in tabun) bonds, but not P–O or P–S bonds. Despite of this, the pH-profile of DFPase does not change as compared to PON1, and the p*K*_a_ is also about 7. It is noteworthy that leaving group of substrates is oriented towards His287 residue (Figure 8b). Thus, it can play exactly the same important role in the protonation of the leaving group as the previously discussed His254 in OPH. For example, the substitution His287Ala leads to a 10-fold decrease in enzymatic activity [55]. Besides, the formed pocket is too small and/or sterically hindered to accommodate, for example, a bulky *p*-nitrophenyl group.

DFPase prefers the *R*_P_-isomer of cyclosarine (the catalytic efficiency is 42 times higher as compared to *S*_P_-isomer), though this difference of activity decreases in the case of a substrate with a less bulky side group (e.g., the difference for sarin stereoisomers is ~10%) [56]. Multiple substitutions of residues (Glu37Ala/Tyr144Ala/Arg146Ala/Thr195Met) formed the pockets for side groups allow a 29-fold increase of catalytic efficiency of DFPase towards *S*_P_-isomer of cyclosarine, while worsening the value of the analogous parameter for *R*_P_-isomer by5.5 times. At the same time, the catalytic efficiency with *S*_P_-isomer of sarin also increases by a factor of 5.5, while it remains approximately at the initial level with *R*_P_-isomer.

Interestingly, though PON1 has an increased free volume in the active site, it also catalytically prefers the *R*_P_-isomer of cyclosarin [57]. It was found that the catalytic efficiency of PON1 towards *S*_P_-isomer of cyclosarin can be improved 135-fold by substitution Leu69Gly/Ser111Thr/His115Trp/His134Arg/Phe222Ser/Thr332Ser.

According to QM and QM/MM calculations [58,59], two transition states with an intermediate in the form of Asp229 phosphorylated by DFP were observed. The phosphorous center has trigonal bipyramidal geometry in this state. Such phosphorylation of Asp229 by DFP was previously established using ^18^O-labeled water [54], thereby confirming the results of calculations. After that, Glu21 is likely to activate the water molecule which destroys this phosphorylation. The energy barriers at the first and second stage were 8.5 and 7.5 kcal/mol [59].

Interestingly, the reaction path was quite different in the case of *S*_P_-isomer of sarin (or soman) [59]. Formation of phosphorylated intermediate was unlikely since it additionally requires ca. 16 kcal/mol as compared with DFP. Even transition from this hypothetical state is energetically hindered too. Overall the single energy barrier was 28.6 kcal/mol and the reaction was thermodynamically unfavorable. Phosphorous center in sarin possessing C–P bonded methyl group seems to be unable to allocate large negative charge of Asp229 and to form some stable intermediate while approaching to each other. Moreover, the possibility of shielding by a methyl group [12] was not studied or revealed. However, the authors suggested direct nucleophilic attack without phosphorylation step by a water molecule activated by Asp229. Exactly this water molecule is normally replaced when DFP is binding to the active center. Thus, there is some flexibility of enzyme mechanism of action which could contribute to their promiscuity towards varying substrates.

Downshifting of PON1 to DFPase by replacement of Asn270 with water [60] has expectedly resulted in an increase of energy barrier with paraoxon (by 50%) and a decrease of it with DFP (by 10%). Most importantly, the reaction with DFP has become thermodynamically favorable in this case. This once again illustrates the great possibilities of gentle transition and fine-tuning of enzyme specificity via genetic modifications.

## 8. Senescence Marker Protein 30

**Figure 11:**
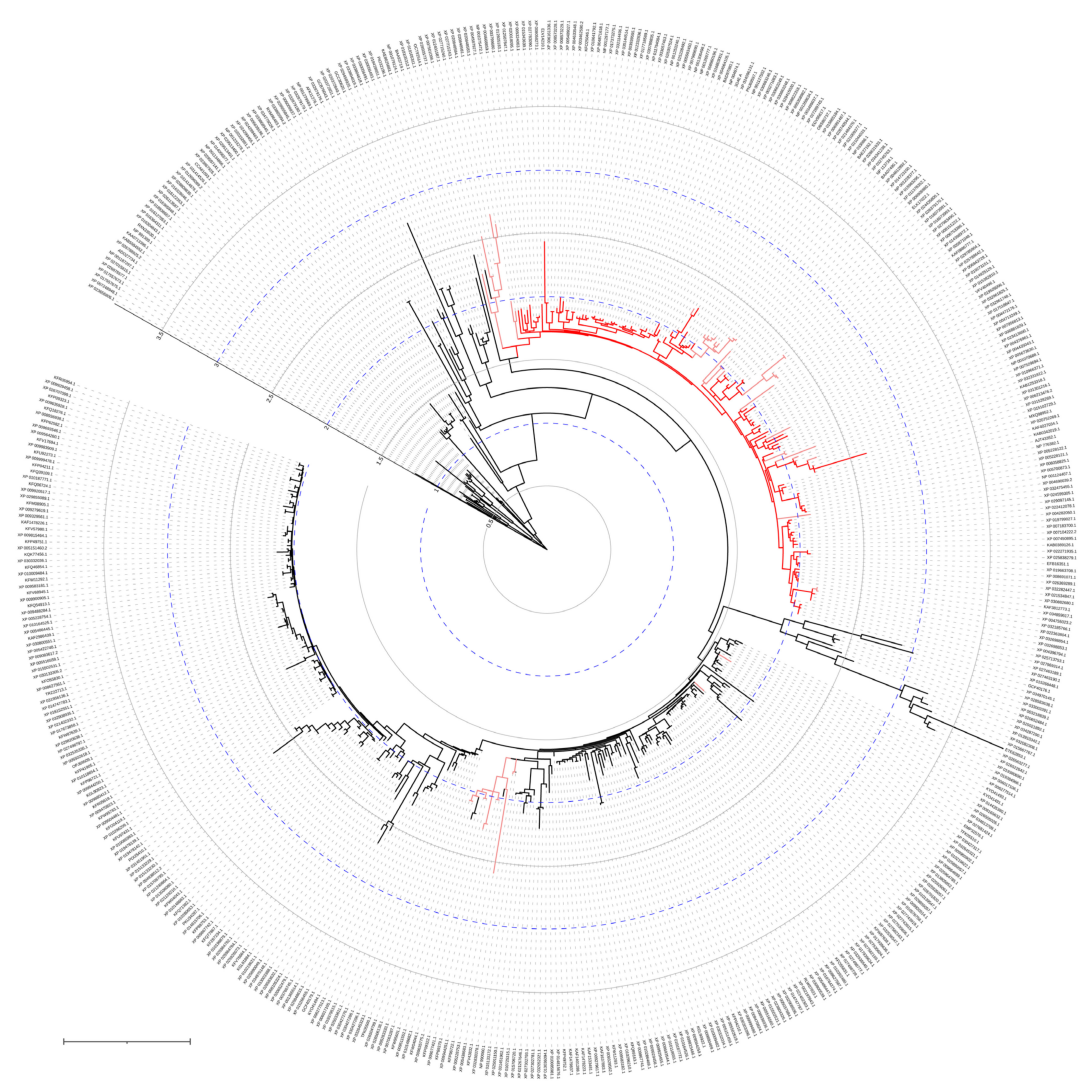
Phylogenetic tree of SMP30 and its homologous enzymes (down to 57% identity). The tree was built as previously with PDB 3G4E as a reference sequence. Enzymes having high (90–100%) and good (80–89%) identity are highlighted with red and pink, respectively.

Senescence Marker Protein 30 (SMP30) was first isolated from mammalian cells as PON1. SMP30 has low amino acid sequence homology with PON1 (~10%) and with DFPase (~20%). However, homologs of this enzyme (Figure 11) appeared to be widespread among both vertebrates and invertebrates [60], and they perform a number of important functions, including participation in the biosynthesis of vitamin C. The protein folds in six-blade β-propeller as PON1 and DFPase (Figure 8c). However, SMP30 lacks a structuring metal ion unlike PON1 and DFPase: a single Asp104 is oriented into this cavity and is unlikely to be sufficient to rigidly fix this ion inside the globule. The catalytic metal ion is coordinated by residues Glu18, Asn154, Asp204, and two water molecules held by Asn103 and Asp104. Replacement of any of these residues with alanine leads to a decrease of activity by a factor of 10 or more [61]. The substrate bound through phosphoryl oxygen completes the coordination sphere of the metal to an octahedron.

The maximal activity of SMP30 is observed with Co^2+^ ions [62], but the Mn^2+^, Mg^2+^, and Zn^2+^ ions have approximately the same efficiency [61]. SMP30 containing Ca^2+^ ions completely loses the OPH-activity that seems to be fundamentally different from PON1 and DFPase.

Interestingly, Ca^2+^ has the weakest binding to the enzyme [61]. Moreover, the pocket of the leaving group is very small as in the case of DFPase, and only small groups (like F^–^) but not large ones (like *p*-nitrophenol) can accommodate it. Accordingly, the number of substrates among OPCs is quite limited for SMP30 [62].

**Scheme 4:**
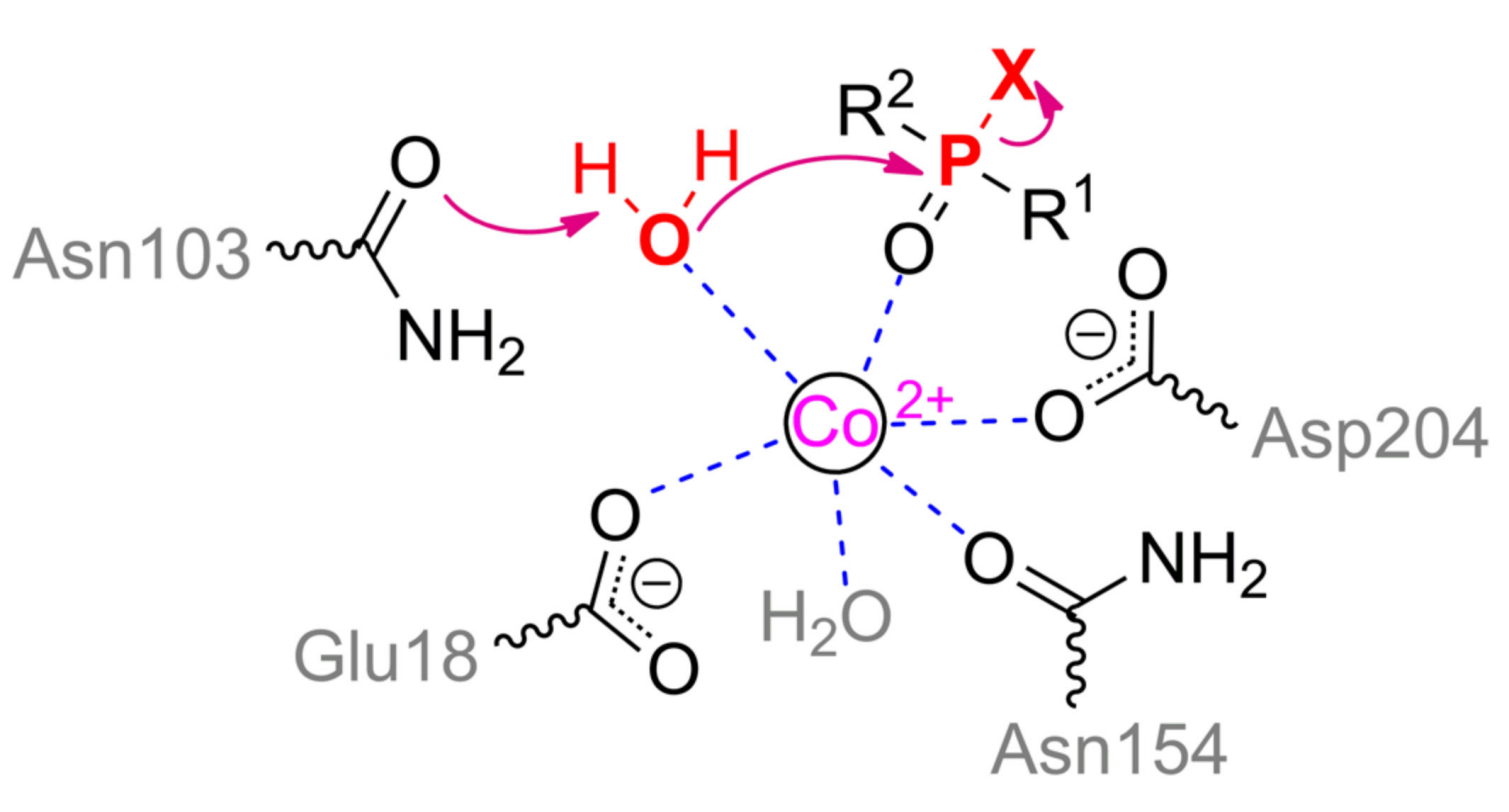
Mechanism of catalytic action of SMP30.

The attacking nucleophile within SMP30 active site is bound directly to the catalytic metal ion as opposed to PON1 and DFPase (Scheme 4), and this is precisely the water molecule that is held by Asn103. A proton can be transferred through Asn103 to Arg101 and then is removed into the solvent medium or interacts with leaving group of substrates. Such nucleophilic attack should be preceded by the destruction of the coordination bond between the activated water molecule and the metal ion, which will be accompanied by a distortion of the coordination complex and by the location of the phosphorus center for an energetically more favorable interaction with the nucleophile. An involvement of arginine (p*K*_a_ = 9.1) in the catalytic act should lead to a shift of the pH-optimum to slightly alkaline conditions as compared to PON1 and DFPase. Indeed, the OPH-activity of SMP30 is usually determined at elevated pH values [62].

## 9. Alkaline and Acid Phosphatases

**Figure 12:**
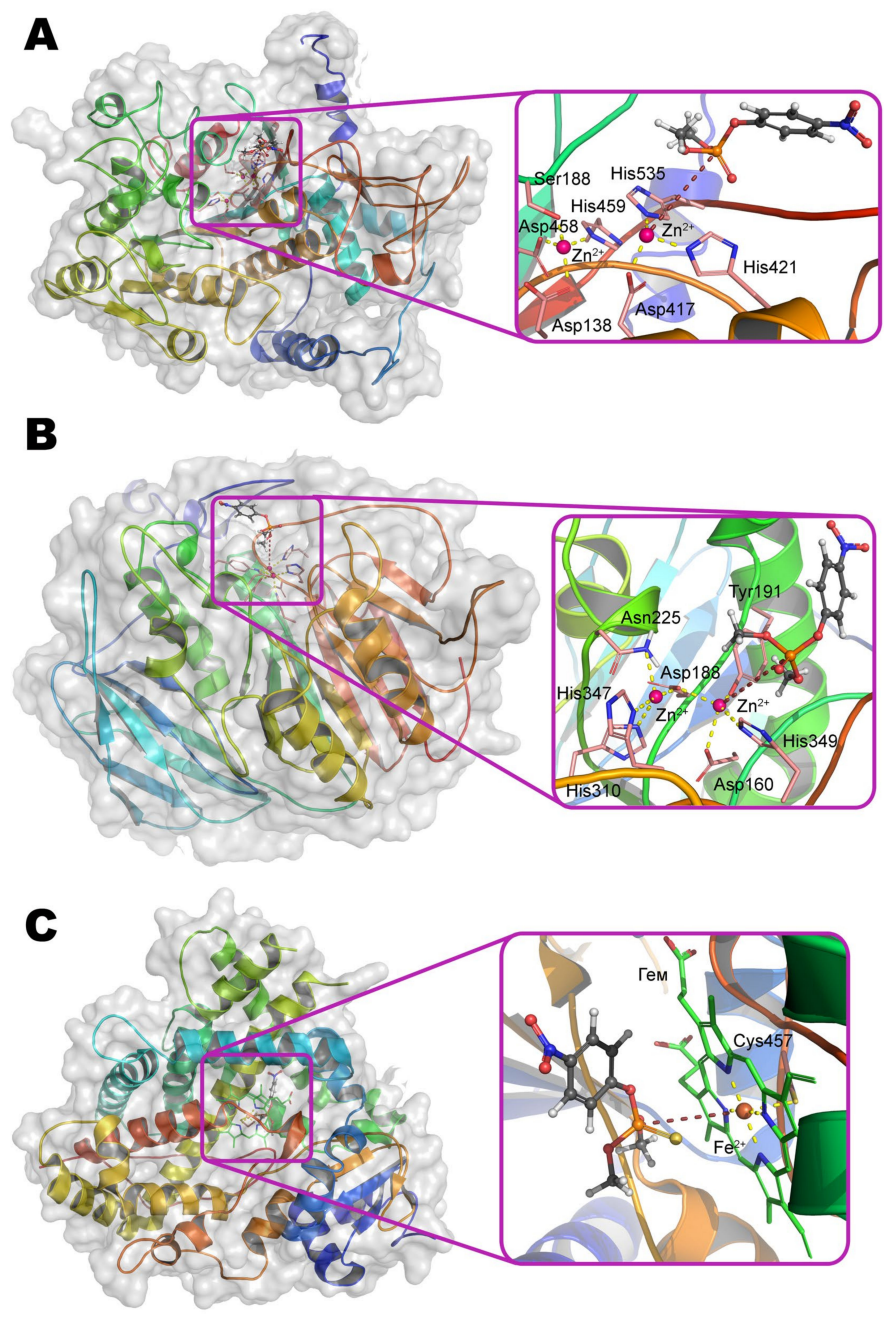
The structure of ALP monomer (**A**), ACP monomer (**B**), and CYP1A1 monomer (**C**) according to data (UniProt A0A2W1BMI5, UniProt Q9MB07, and PDB 4I8V, respectively). The entrance to the active centers of the enzymes is framed, and their structures are shown in detail within insets. The structures of ALP and ACP were predicted with I-TASSER server (http://zhanglab.ccmb.med.umich.edu/I-TASSER/) as described in [64]; after that the residues of the active centers were aligned with known structural homologues (PDB 1K7H and 1KBP, respectively), and coordinates of metal ions were determined and used further. The binding of the substrate methyl paraoxon (**A**,**B**) and methyl parathion (**C**) was determined as previously. The coordination bonds of metal ions with amino acid residues in active centers, as well as with labile groups of the substrate are shown by a dashed line.

**Figure 13:**
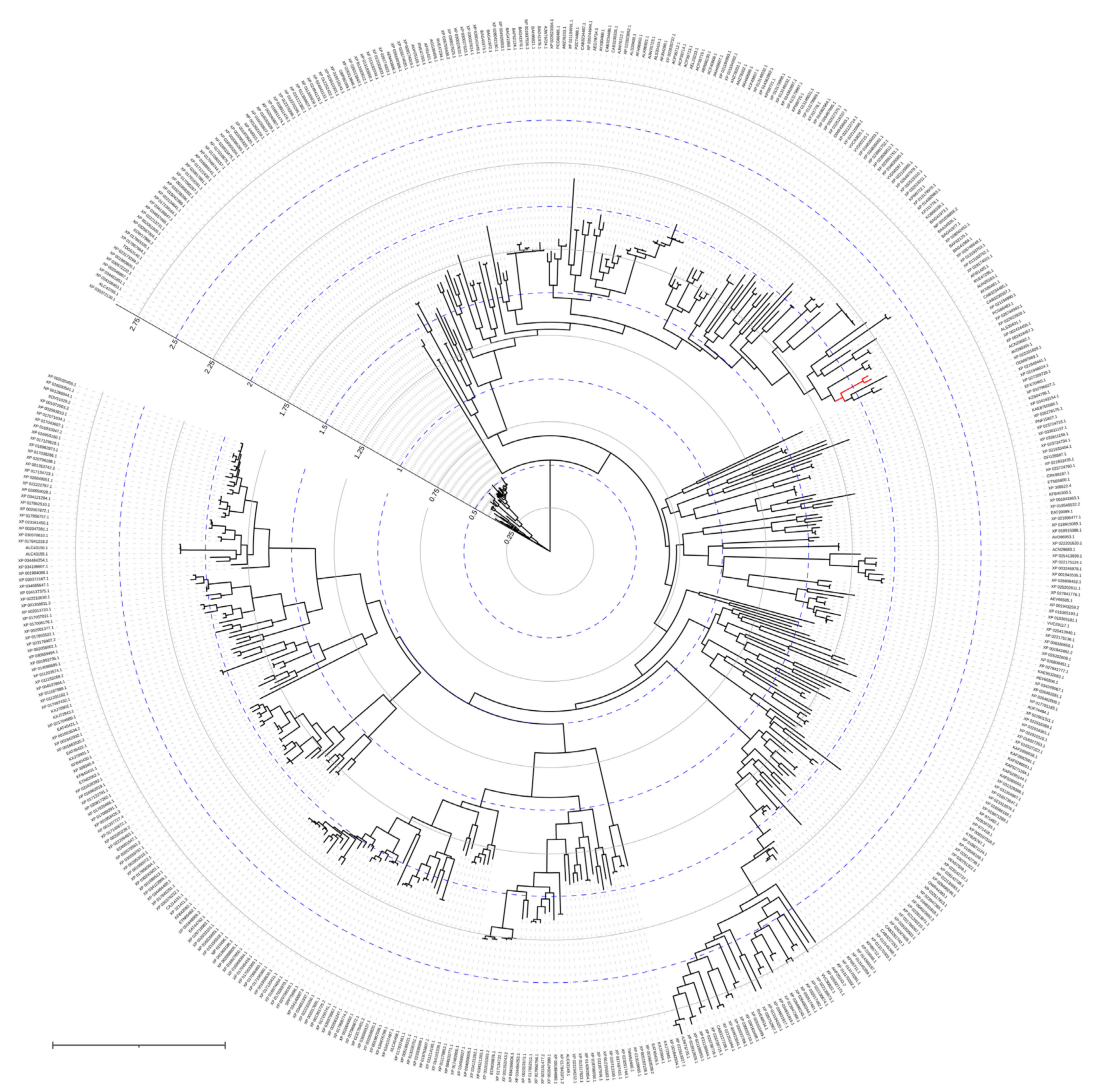
Phylogenetic tree of ALP and its homologous enzymes (down to 46% identity). The tree was built as previously with UniProt A0A2W1BMI5 as a reference sequence. Enzymes having high (90–100%) identity are highlighted with red.

**Figure 14:**
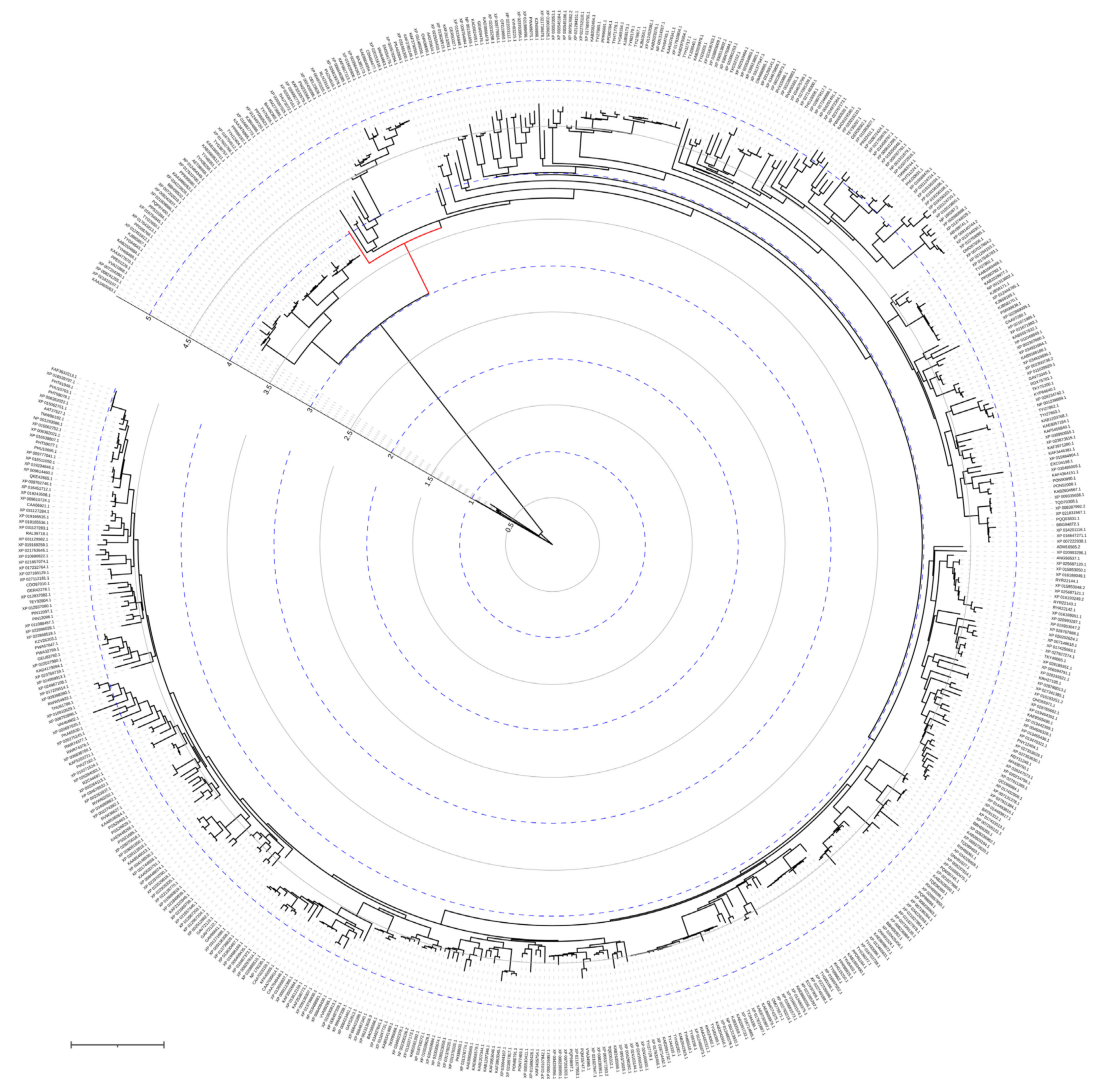
Phylogenetic tree of ACP and its homologous enzymes (down to 40% identity). The tree was built as previously with UniProt Q9MB07 as a reference sequence. Enzymes having high (90–100%) identity are highlighted with red.

Alkaline (ALP) and acid (ACP) phosphatases are homodimers and are widespread among living organisms and catalyze the hydrolysis of OPCs in the form of monoesters and rarely diesters (Figure 12). Their activity towards triesters which are mainly used as pesticides and chemical warfare agents is usually extremely low. It allows the use of these enzymes in the analysis of OPCs via competitive inhibition of reaction with usual substrates [63]. Nevertheless, active variants of ALP and ACP acting on triesters are often isolated from insect pests and plants that have evolved resistance to the pesticides used, including due to these enzymes. As a result of this accelerated evolution (Figure 13 and Figure 14), the resulting variants (being predicted in a similar procedure with [64]) inherit the main structural motif of their precursor proteins (Figure 12a,b).

ALP and ACP (in particular, the metal-dependent variants) typically contain 3 and 2 metal ions in their active site, respectively. The third metal ion is considered not to be directly involved in catalysis and rather performs a structuring and auxiliary function while increasing the positive charge in the active center and stabilizing the enzyme against the inactivating effect of elevated temperatures. The maximal activity is observed with Zn^2+^ and Mg^2+^ [65,66], but some activity can be retained with Mn^2+^, Ca^2+^, Co^2+^, Mo^2+^, and Cu^2+^.

ALP is a founder enzyme of the superfamily of the same name with a typical sandwich αβα folding. ACP, in turn, forms a distinct clade in the superfamily of histidine-containing phosphatases. As a result, their mechanism of action has both similar and different elements.

**Scheme 5:**
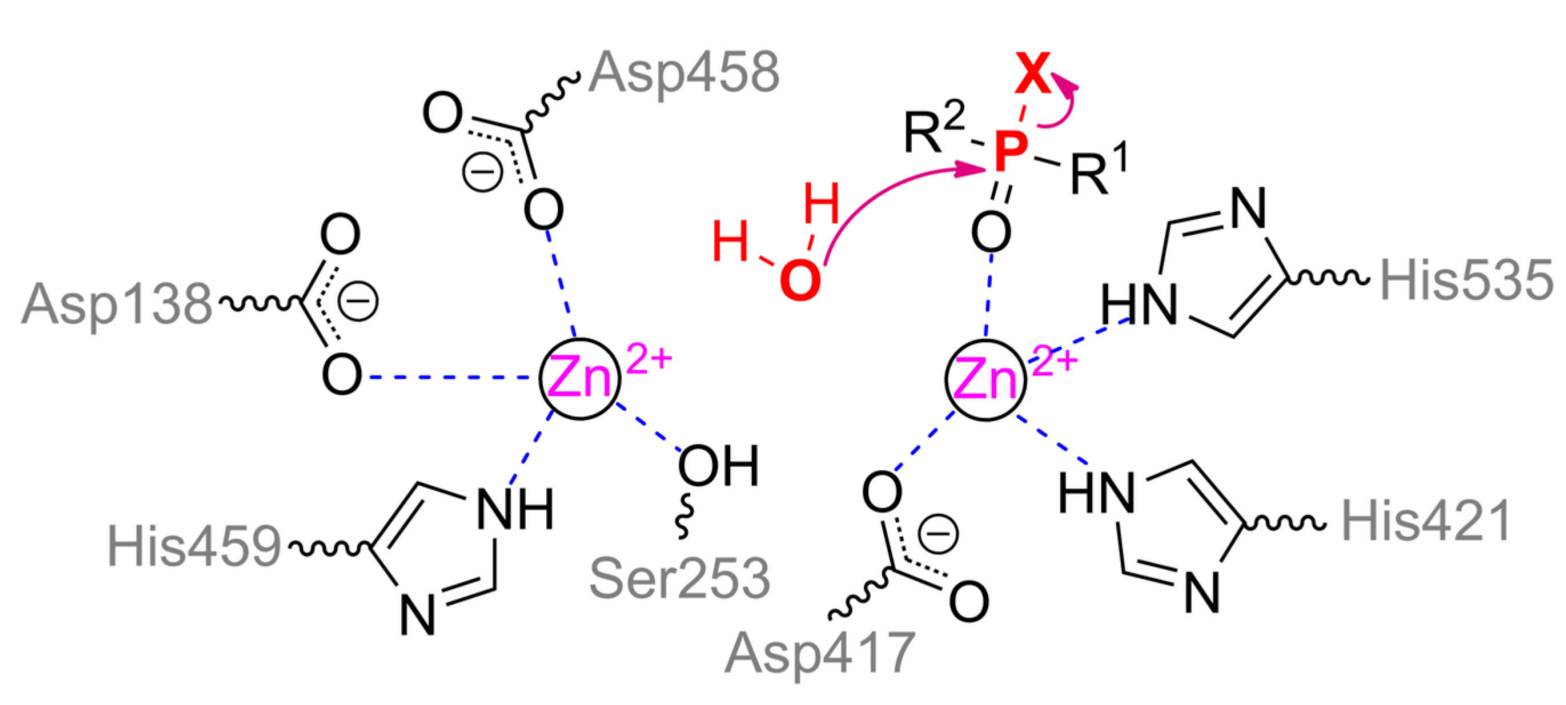
Mechanism of catalytic action of ALP.

α-metal ion of ALP from moth *Helicoverpa armigera* is coordinated by residues Ser188, Asp458, His459 and is coupled via a bridging Asp138 to the structuring metal ion, which, in turn, is adjacent to residues His251, Ser253, and Glu412. Thus, the coordination sphere of both metals is a tetrahedron. β-metal ion is more exposed to the solvent and is also tetrahedrally coordinated by residues Asp417, His421, His535, and water molecule (Scheme 5). According to the experimental and computational mechanism [67], the monoester substrate binds to the β-metal by one of its oxygen, while other oxygen replaces serine from the coordination sphere of the α-metal. Then this residue carries out a nucleophilic attack on the phosphorus center while forming a phosphorylated derivative. However, such binding with the α-metal is impossible in the case of the triesters of OPCs. Moreover, side substituents at the phosphorus center sterically hinder deep penetration of the substrate into active center to realize the mechanism observed for OPH. As a result, ALP is most likely to have a mechanism similar to SMP30, when the same metal ion binds the substrate and activates the water molecule for nucleophilic attack. This assumption is supported by the shift of pH-optimum to neutral values while changing a substrate from *p*-nitrophenyl phosphate to methyl paraoxon [65].

**Scheme 6:**
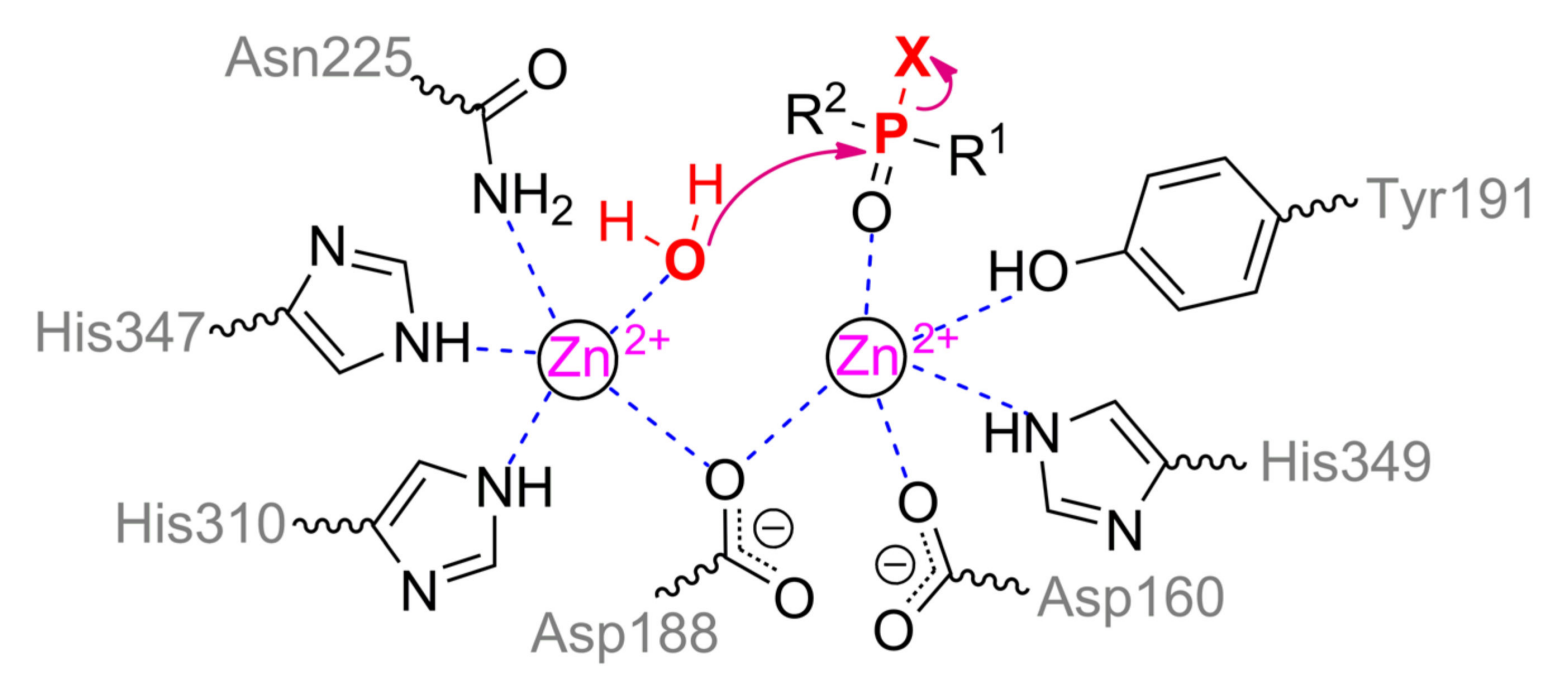
Mechanism of catalytic action of ACP.

The structure of the binding site of metal-dependent ACP has common features with OPH (Figure 12b). α-metal ion is coordinated by residues Asn225, His310, and His347. β-metal ion is more exposed to the solvent and coordinated by residues Asp160, Tyr191, and His349. Asp188 and a water molecule are the bridging ligands. Thus, the coordination sphere of both metals is a trigonal bipyramid (Scheme 6). To additionally correct an orientation of substrate, two histidine residues are located in a close vicinity and are able to compensate the charge on the oxygen atoms at the phosphorus center (however, only a single His226 is present in the enzyme from duckweed *Landoltia punctata*, having an OPH-activity).

According to the classical mechanism of ACP, the monoester substrate binds through phosphoryl oxygen to the β-metal and replaces a water molecule from the coordination sphere. Then this activated water molecule carries out a nucleophilic attack on the phosphorus center in full accordance with the OPH mechanism. However, this mechanism is difficult to realize with the triesters of OPCs as in the case of ALP, and catalysis is carried out by single metal.

Interestingly, based on the pH-profile with *p*-nitrophenyl phosphate, the p*K*_a_ of a catalytically important group is approximately 4 [66]. This value corresponds to the p*K*_a_ of the side group of aspartic acid, one of which is a bridging ligand between metals and is obviously critical for the correct assembly of the active site. Besides, ACP was found [66] to be capable of catalyzing the hydrolysis of P–O and, to a lesser extent, P–S bond, but cannot act on the C–P bond (e.g., in trichlorfon, *O*,*O*-dimethyl (2,2,2-trichloro-1-hydroxyethyl)phosphonate).

## 10. Cytochrome

Cytochromes are enzymes belonging not to hydrolases, but to oxidoreductases that are capable of catalyzing the oxidation of OPCs. In general, cytochromes are very often recruited within the body to detoxify various toxicants and/or xenobiotics [10,68]. Therefore, their effect on OPCs can be nonspecific, and their activity can be greatly reduced in comparison with other substrates or even inhibited by OPC alone [69] or in combination with other substances [70]. Nevertheless, compounds of a wide subclass of OPCs containing P=S bond (e.g., parathion [71], phosmet [72], diazinon [73], etc.) are designed specifically for biochemical activation due to cytochromes. As a result of the reaction, a product with P=O bond is formed, which greatly increases the toxicity of the initial active substance.

**Figure 15:**
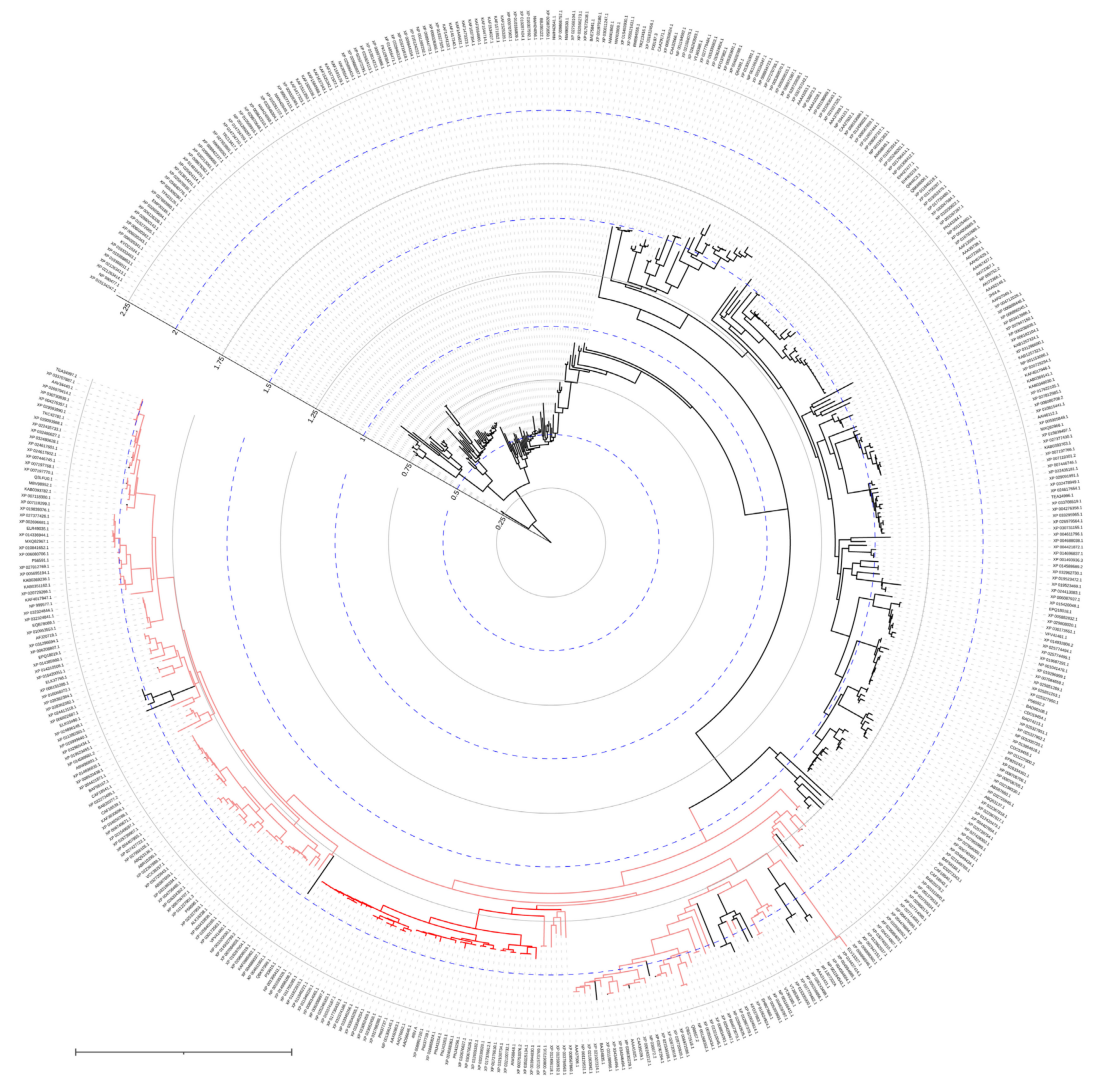
Phylogenetic tree of CYP1A1 and its homologous enzymes (down to 63% identity). The tree was built as previously with PDB 4I8V as a reference sequence. Enzymes having high (90–100%) and good (80–89%) identity are highlighted with red and pink, respectively.

Cytochrome P450 is a typical representative of the heme-containing enzyme superfamily (Figure 12c), and beginning with marsupials a human isoform CYP1A1 is a quite typical enzyme among mammals with few exceptions (Figure 15).

**Scheme 7:**
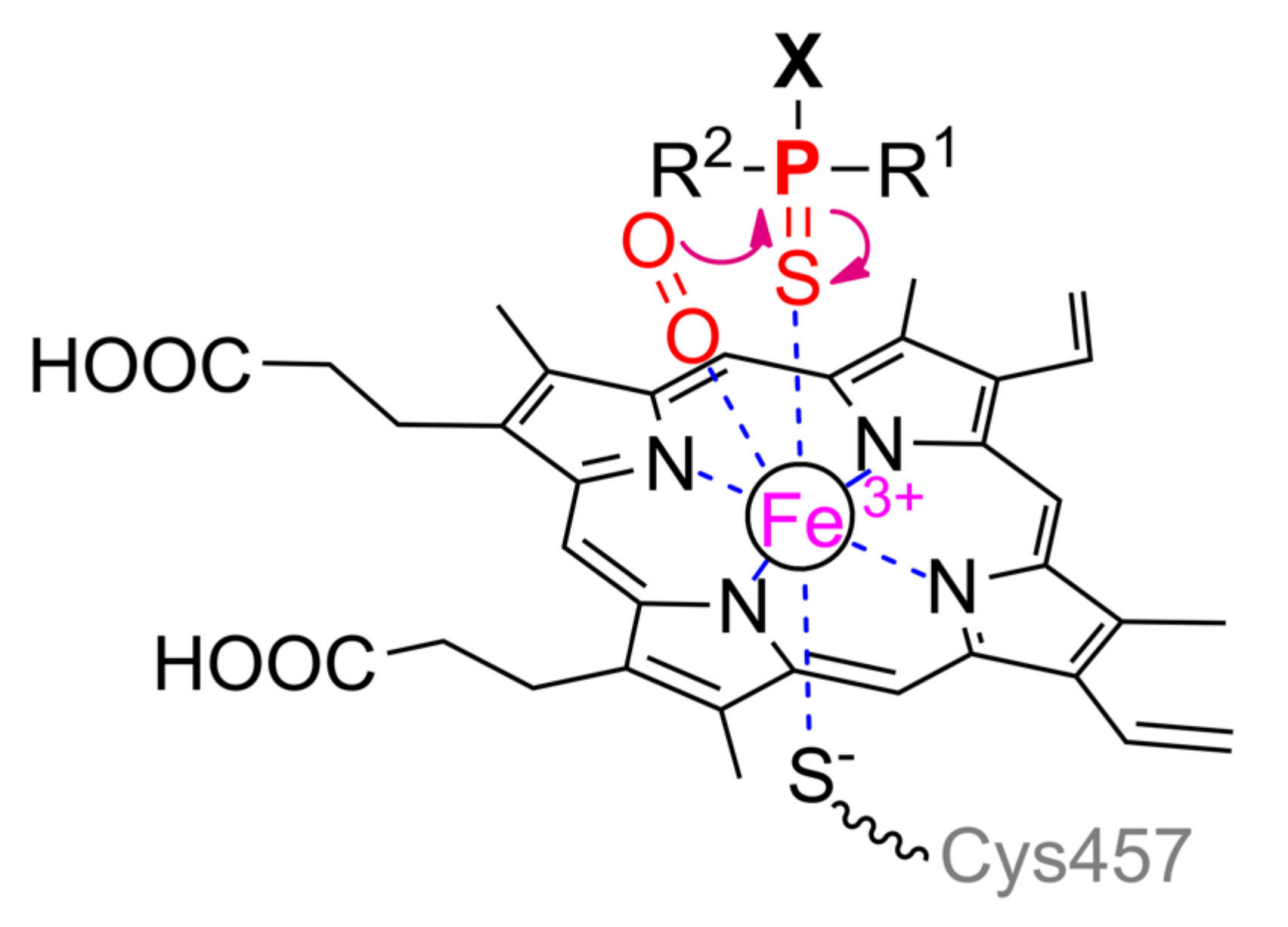
Mechanism of catalytic action of CYP1A1.

The Fe^2+^ ion in the center of the porphyrin macrocycle binds to an amino acid residue, for example, cysteine, on the opposite side of this plane (Scheme 7). Molecular oxygen is coordinated with the same ion from the other side, resulting in its activation. Even polycyclic compounds can penetrate into the large cavity of the active center; therefore, substrates of moderate or small sizes will be too mobile during binding. To avoid this, the substrate binding leads to conformational changes in the protein globule from an open to a closed form [74], thereby preventing the release of a small substrate from the active center. A peroxy radical attacks the phosphorus center with subsequent migration of the radical to the sulfur atom, intramolecular rearrangement, and cleavage of the P–S bond.

Such peroxy radical is also formed in the case of another heme-dependent oxidoreductase, chloroperoxidase from fungus *Caldariomyces fumago*, when hydrogen peroxide is added to the reaction medium. This enzyme is capable of oxidizing sulfur-containing OPCs as cytochromes [75]. Though such metabolic activation leads to an increased toxicity of the reaction product as compared to the initial compound, on the whole, it can be considered positive due to the fact that the activity of other hydrolytic enzymes discussed earlier is much higher towards such oxidized OPCs.

In addition to sulfur-containing OPCs, aromatic substituents can be modified by cytochromes also [76]. According to QM-simulation, the largest energy barrier for the first monohydroxylation was in the *para*-substitution of phenyl substituent within triphenyl phosphate, and the least one was in *ortho*-position. The second hydroxylation could even lead to modification of *ipso*-position, followed by degradation of P–O bond. That is, some proper OPCs can be degraded via sequential oxidative hydroxylation by cytochromes.

## 11. Inversion of Inhibition to Biocatalysis: Cholin- and Carboxylesterases

**Figure 16:**
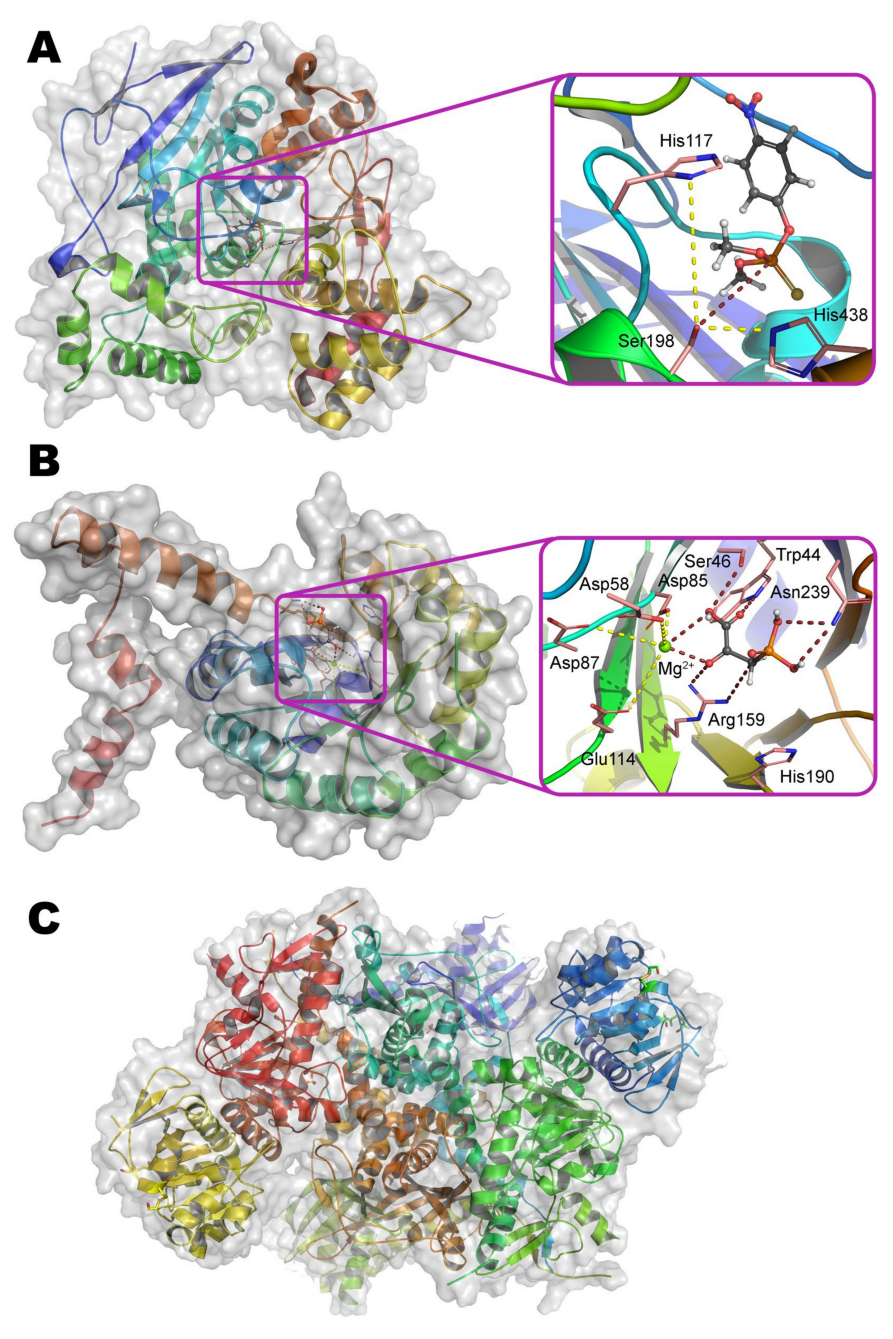
The structure of modified AChE (**A**), PEPP (**B**), and eight-subunit complex of C-P lyase (**C**) according to X-ray diffraction data (PDB 2XMG, 1S2V, and 4XB6, respectively). The entrance to the active centers of the enzymes is framed, and their structures are shown in detail within insets. The binding of the substrate methyl parathion (**A**) and phosphonopyruvate (**B**) was determined as previously. The coordination bonds of metal ions with amino acid residues in active centers, as well as with labile groups of the substrate are shown by a dashed line.

**Scheme 8:**
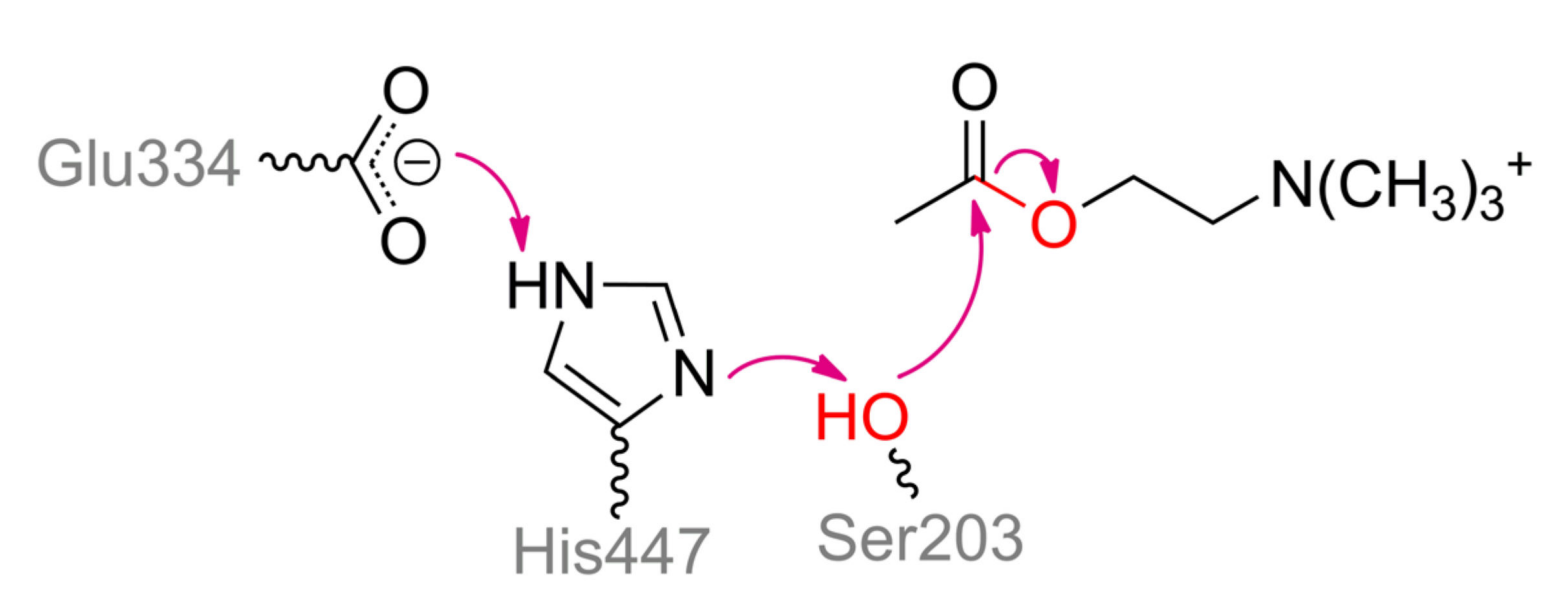
Mechanism of catalytic action of human AChE.

**Figure 17:**
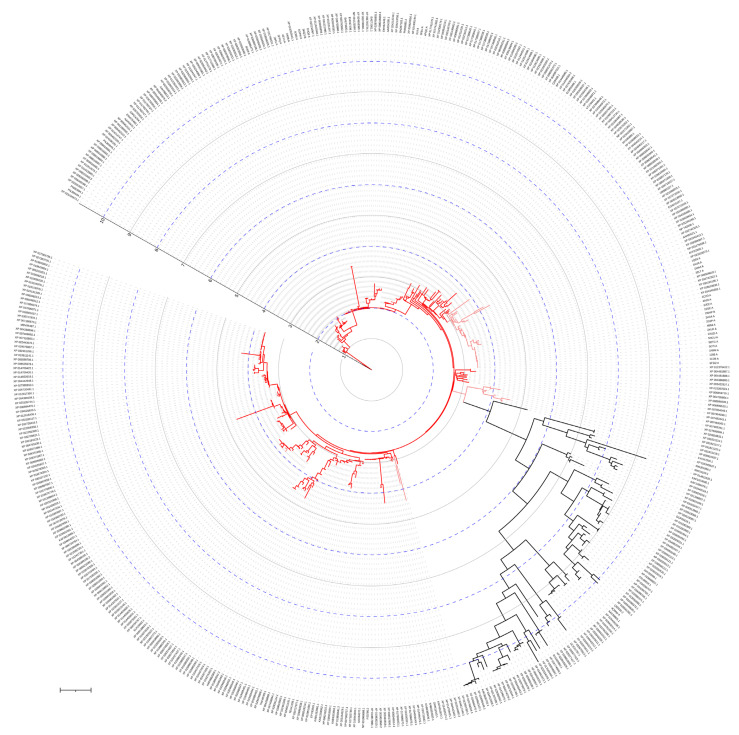
Phylogenetic tree of human AChE and its homologous enzymes (down to 58% identity). The tree was built as previously with PDB 2XMG as a reference sequence. Enzymes having high (90–100%) and good (80–89%) identity are highlighted with red and pink, respectively.

As it was mentioned earlier, OPCs are designed to inhibit serine hydrolases, including cholin- and carboxylesterases [77]. Nevertheless, it is possible to tune on their activity with OPCs while genetically modifying these enzymes. Cholin- and carboxylesterases are divided into several families but have a typical (α/β)_8_ fold being common for alpha/beta hydrolase superfamily (Figure 16a). Their catalytic centers vary significantly in size and shape, depending on natural substrates geometry. However, they share a similar catalytic triad Ser–His–Glu(Asp) (Scheme 8). Normally His promotes nucleophile (Ser) to attack substrate giving a carboxylated serine intermediate, while its strength is insufficient to initiate dephosphorylation of Ser. Thus, most researchers concentrate their efforts on genetic modification of the microenvironment of His and other amino acid residues. However, this artificial progress is now rather modest [78], and natural evolving is much more creative [79,80] (Figure 17).

It should be noted here that a contribution of geometries of phosphorylated adducts to the rate constants appears to be underestimated. According to the isotope effect on AChE, bulkier substituents decrease rate constants dramatically [81].

Additionally, a lot of OPCs have ester bond(s) within substituents of phosphorous (e.g., malathion), and thus carboxylesterases can catalyze hydrolysis in this site [82].

## 12. Enzymes Acting on Phosphonates

This group of enzymes is highly diverse and wide, but their activity towards phosphonates unites all of them. C–P bond in phosphonates such as methylphosphonic acid is extremely stable. Thus, it is not possible to break it under reasonable conditions otherwise than via enzymatic treatment, i.e., with additional preliminary modification(s).

**Figure 18:**
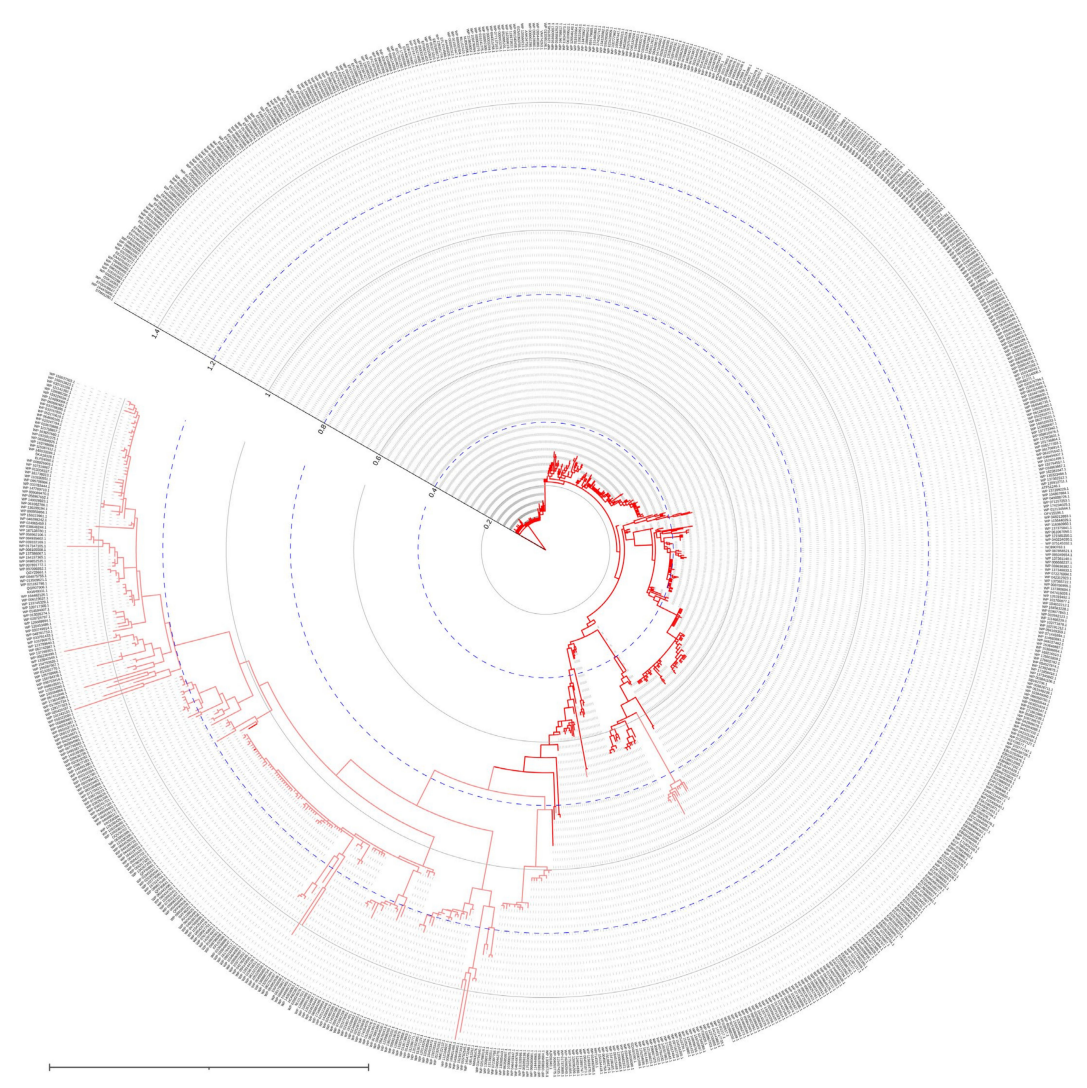
Phylogenetic tree of PhnJ subunit of C-P lyase and its homologous enzymes (down to 85% identity). The tree was built as previously with PDB 4XB6 as a reference sequence. Enzymes having high (90–100%) and good (80–89%) identity are highlighted with red and pink, respectively.

C–P lyase is one of the most famous and interesting enzymatic complex [83]. For a long time, it was impossible to isolate and purify this “enzyme,” and its activity was investigated exclusively within bacteria [84]. When it was isolated and crystallized for the first time, it was found to consist of 8 subunits (while its gene cluster encodes 14 proteins totally) (Figure 16c). The key subunit (PhnJ) is highly conserved and distributed widely among microorganisms (Figure 18).

**Scheme 9:**
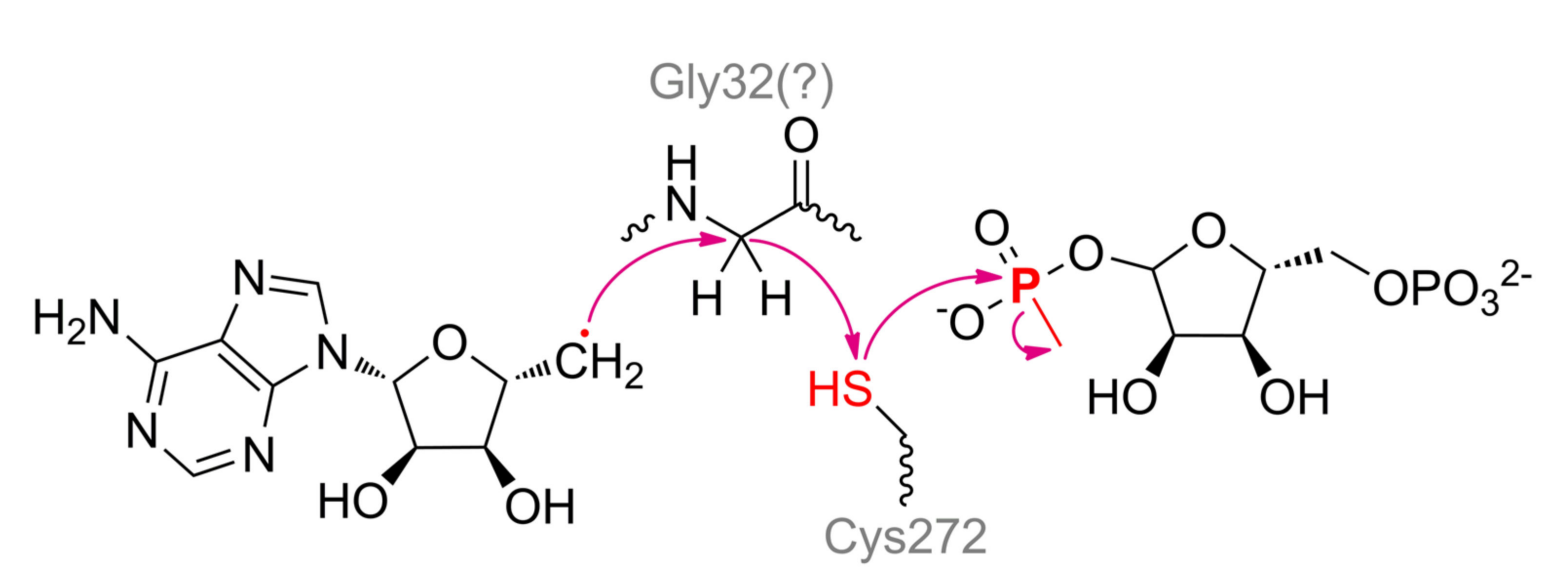
Mechanism of catalytic action of PhnJ.

There are some difficulties in classifying PhnJ to a certain enzyme family now [85], and it temporarily forms a distinct group of its own. Using 4Fe–4S cluster and *S*-adenosyl methionine as a co-substrate, PhnJ catalyzes radical cleavage of activated intermediate (5-phosphoribosyl-1-phosphonate) to methane and 5-phosphoribosyl 1,2-cyclic phosphate [86], followed with decyclization via hydrolysis by a phosphoribosyl cyclic phosphodiesterase, homodimeric PhnP. The 4Fe–4S cluster binds with Cys241, Cys244, and Cys266 at the C-terminus of PhnJ, while Cys272 realizes the radical attack on the phosphorous (Scheme 9). There is a prevailing opinion that a glycyl radical formed by *S*-adenosyl methionine from Gly32 activates the Cys272 via an analogous mechanism to pyruvate formate-lyase [87] (it has less than 15% identity, and Cys positions are not conserved). However, the distance between Gly32 and Cys272 is ca. 30 Å and it is too long as compared with ca. 4 Å within pyruvate formate-lyase. Moreover, Gly32 is buried in the interface with PhnH when C–P lyase complex forms. Even Gly147 of another PhnH subunit (localized at the C-terminus) and/or Gly7 of PhnI subunit (localized at the N-terminus) are/is much closer (at 16–17 Å), while Gly72 and Gly245 of the same PhnJ are the closest (at 9–10 Å). Such discrepancy between isotope labeling experiments and structural analysis is somehow confusing and could be caused by inadequate conditions (e.g., PhnJ subunit was used alone and as a part of GST-fusion for kinetic measurements) or erroneous assembling of C–P lyase complex. Yet another point highlighted by Manav et al. [88] is a possibility of structural rearrangements in the presence of Zn^2+^ ions used instead of intact 4Fe–4S cluster. Thus, additional investigations are required.

Interestingly, phosphomethylpyrimidine synthase (i.e., another radical enzyme dependent on *S*-adenosyl methionine of TIM barrel fold) is likely to be an ‘improved’ version of PhnJ in spite of the fact it has less than 10% identity but conserves all four cysteine residues at the C-terminus to accommodate 4Fe–4S cluster [89]. Moreover, elongated C-terminal domain of ca. 60 Å allows to lid the active site of another subunit located at the same distance, thus resulting in a close proximity of 4Fe–4S cluster with radical donor and reaction to proceed.

**Figure 19:**
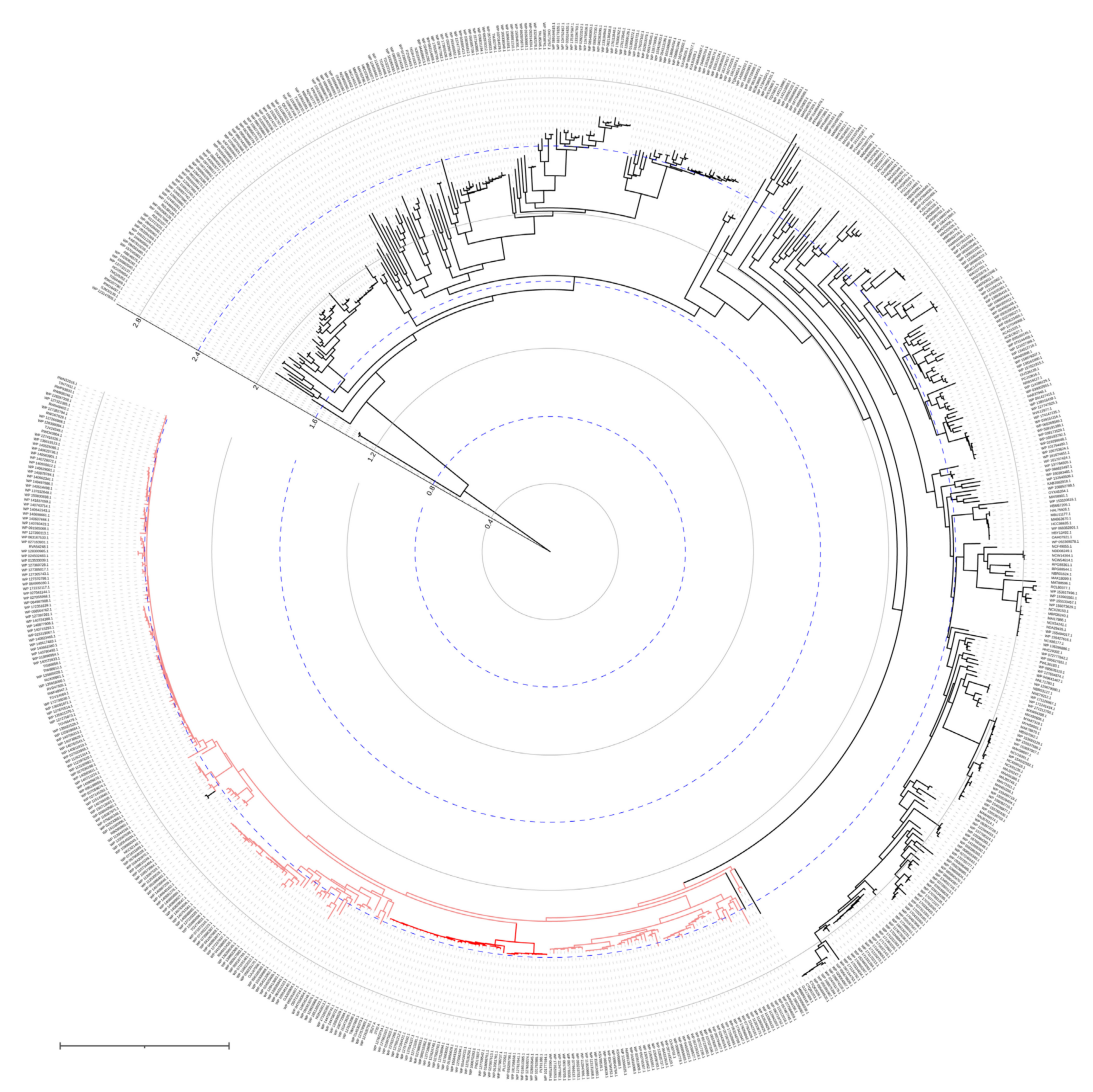
Phylogenetic tree of PhnA and its homologous enzymes (down to 44% identity). The tree was built as previously with PDB 3SZY as a reference sequence. Enzymes having high (90–100%) and good (80–89%) identity are highlighted with red and pink, respectively.

**Scheme 10:**
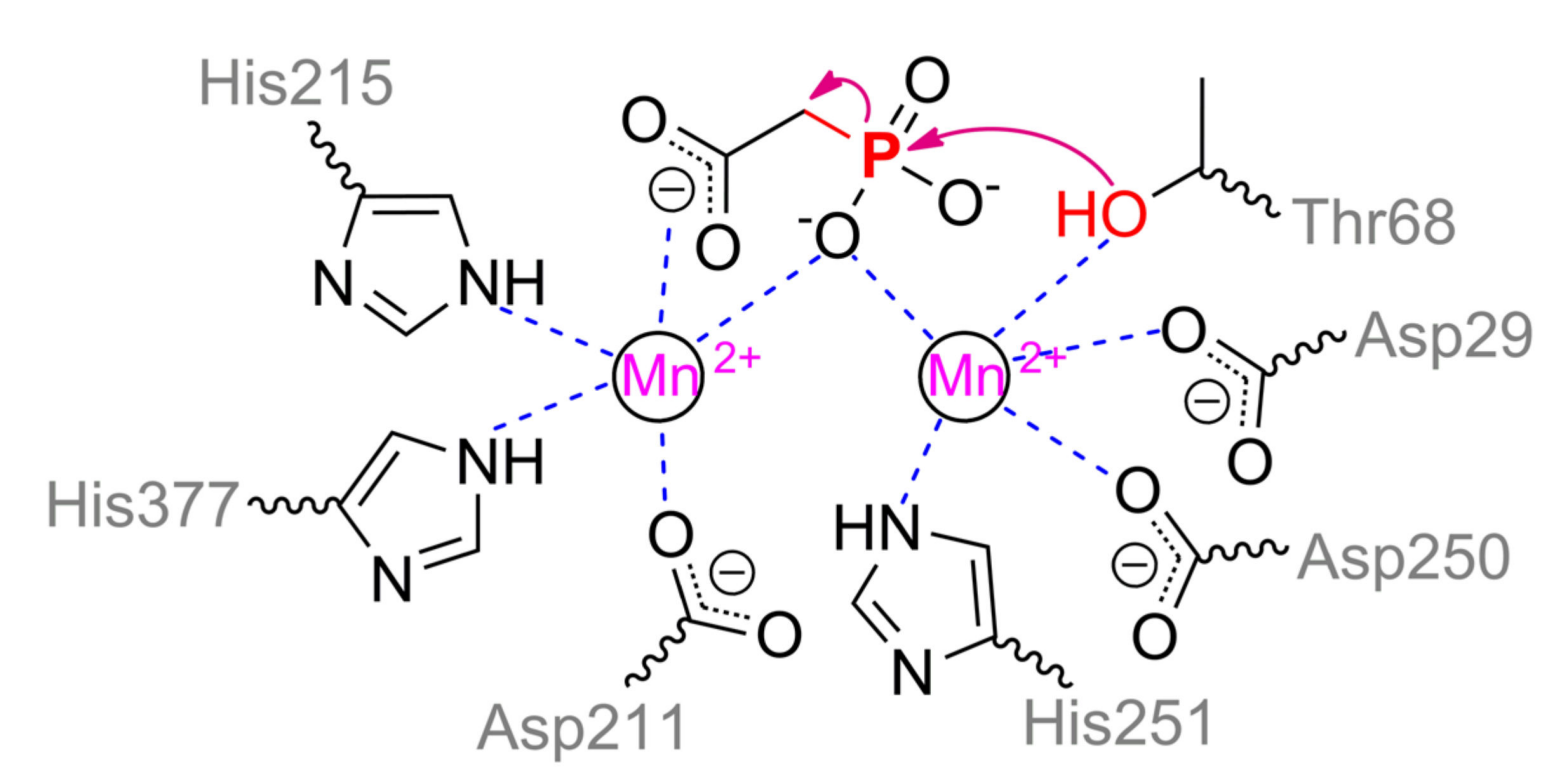
Mechanism of catalytic action of PhnA.

Phosphonoacetate hydrolase (PhnA), for example, from *Sinorhizobium meliloti*, is another interesting and widely distributed enzyme (Figure 19). PhnA belongs to the superfamily of alkaline phosphatase and folds similarly, excepting a curious capping domain [90]. PhnA contains only two metal ions in the active center versus three in ALP. The greatest activity is in the case of Fe^2+^ and Mn^2+^, while the worst one is with Mg^2+^. The coordination sphere of the more buried α-metal ion includes Asp29, Thr68, Asp250, His251, and water molecule could complete a trigonal bipyramidal geometry (Scheme 10). β-metal ion has a tetrahedral geometry and is slightly more exposed to the solvent, while it is surrounded by Asp211, His215, His377, and water molecule. The last one is replaced when substrate is binding with its carboxylic group. Moreover, geometry of β-metal ion seems to be changed to the same trigonal bipyramid in the intermediate state. Interestingly, though one of substrate oxygens is coordinated by both metals a little bit asymmetrically in this state, it appears to be sufficient to compensate its strong negative charge. Another interesting and important point is a binding of intermediate directly with Thr68 (i.e., some sort of phosphorylation). This is not new and other serine hydrolases such as AChE are also capable to interact in a similar way. However, as described earlier such interaction with AChE is possible through ‘prepared’ serine by proton transfer via classic triad Ser–His–Glu(Asp). There is no such possibility in the PhnA. For PhnA from both *Sinorhizobium meliloti* [90] and *Pseudomonas fluorescence* (it has 52% identity) [91], modulation by coordinated metal ion was proposed. However, p*K*_a_ of 6.4 for catalysis to occur unambiguously determines the histidine(s). The only available residues are His294 and His295 (equal to His285 and His286 for enzyme from *Pseudomonas fluorescence* cells) near the entrance to the active center and point mutations of both to alanine lead to a decrease of catalytic constant (up to 8-fold) [91]. Thus, proton could be transferred through a chain Thr68–water–Lys130(Lys132)–Tyr181––His294(His295) and/or Thr68–Asn69–Asn89–Asp289–Tyr291––His294(His295). Interruption of any of this chain at Lys130/Lys132 or Asn89 results in a dramatic drop of enzyme activity (up to complete inactivation). One more interesting question is the fate of the leaving group. Nucleophilic attack by Thr68 could produce an acetoxy carbanion being unfavorable chemically [86] and energetically under reaction conditions. At the same time, lysines are known to perfectly stabilize such carbanions [92]. Lys130 is correctly oriented to intercept the carbanion while Lys132 could coordinate its carboxy group. Backward transfer of the proton from His294(His295) releases the product, and thus it may be an alternative explanation of observed p*K*_a_ and roles of histidines. It should be noted that PhnA from *P*.*fluorescence* also accepts other substrates such as phosphonocarboxylic acids, phosphonoaldehydes, and even usual ALP substrate – *p*-nitrophenylphosphate [91].

**Scheme 11:**
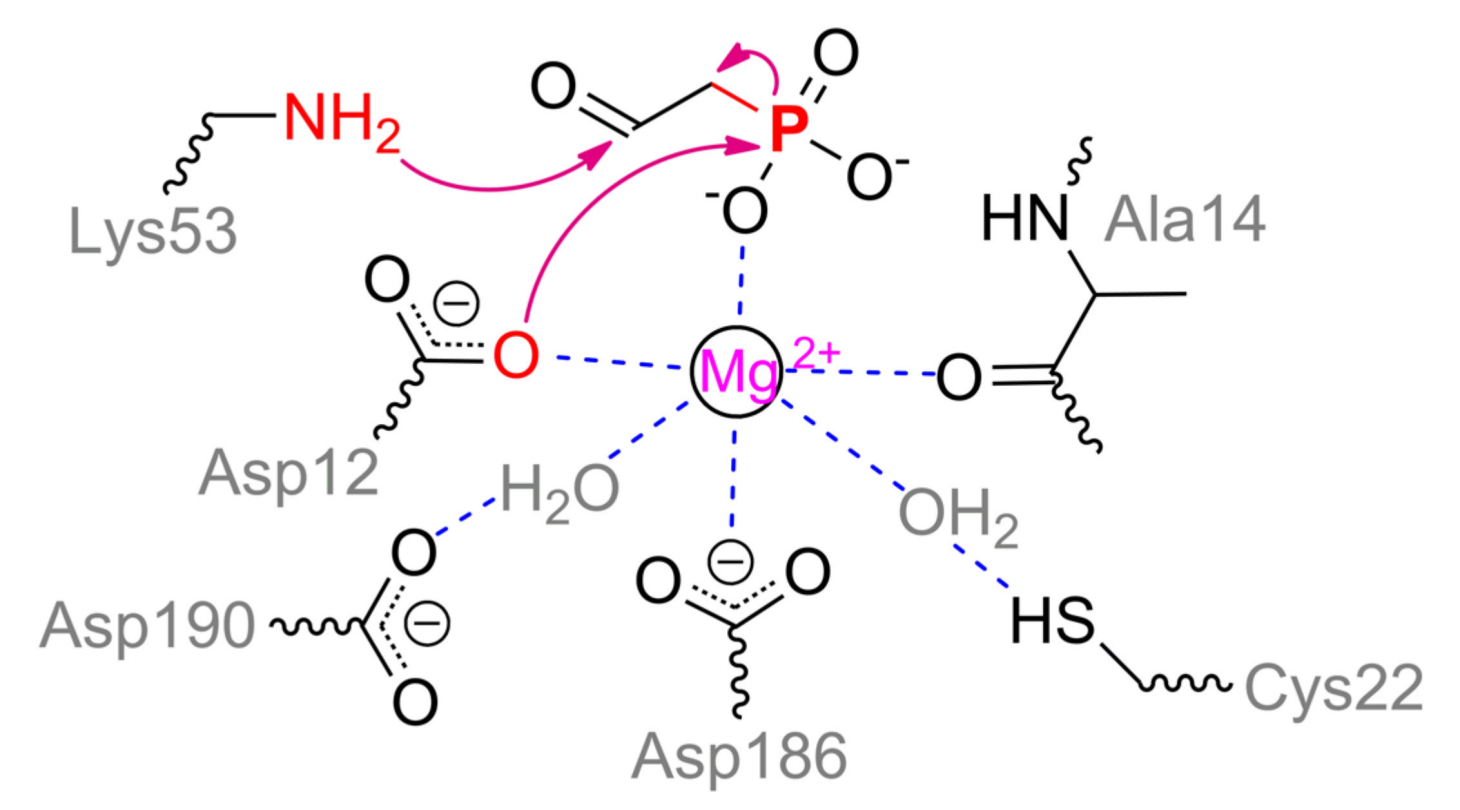
Mechanism of catalytic action of PhnX.

PhnA utilizes the same phosphonoaldehydes which are conventional substrates of phosphonoacetaldehyde hydrolase (phosphonatase, PhnX) [93]. PhnX belongs to a haloacid dehalogenase superfamily of enzymes and has been thoroughly investigated to date [86]. It participates in phosphorous acquisition during metabolism of 2-aminoethylphosphonate which preliminary is treated with transaminase PhnW to produce phosphonoacetaldehyde. PhnX contains a single Mg^2+^ ion coordinated by Asp12, Asp186, water molecule (held by Asp190) and distal oxygen of Ala14 (numeration for enzyme from *Bacillus cereus*, PDB 1RDF) (Scheme 11). During substrate binding and enzyme rearrangement to a closed form, phosphonyl oxygen and water molecule held by Cys22 complete an octahedral coordination sphere. The second phosphonyl oxygen of substrate is coordinated by Arg160, and the last one is very likely to interact with Thr126. The key residue is Lys53 which forms a Schiff base with acetaldehyde group. Such a base is multiply stabilized in a closed enzyme form [86] that facilitates proper departure of ‘hard’ carbanion during catalysis. Asp12 is generally acknowledged to perform a nucleophilic attack on the phosphorous center that stimulates cleavage of C–P bond. Further, a doubly modified enzyme should regenerate its initial state. For that, two processes are realized simultaneously: water molecule activated together by Tyr128, Met49, and His56 attacks a Schiff base, releasing acetaldehyde; water molecule generated during Schiff base formation attacks phosphorous center to produce phosphate. The same Schiff base was proposed to activate the latter [86]. However, some proper quadrangle bonding may be needed for a distal methyl group as is the case for acylphosphatase [94]. The same phosphate generation can be achieved via attack by water already coordinated/activated with metal and Cys22 in the upper plain. That could result in the same isotope stereoconfiguration in phosphate product.

**Figure 20:**
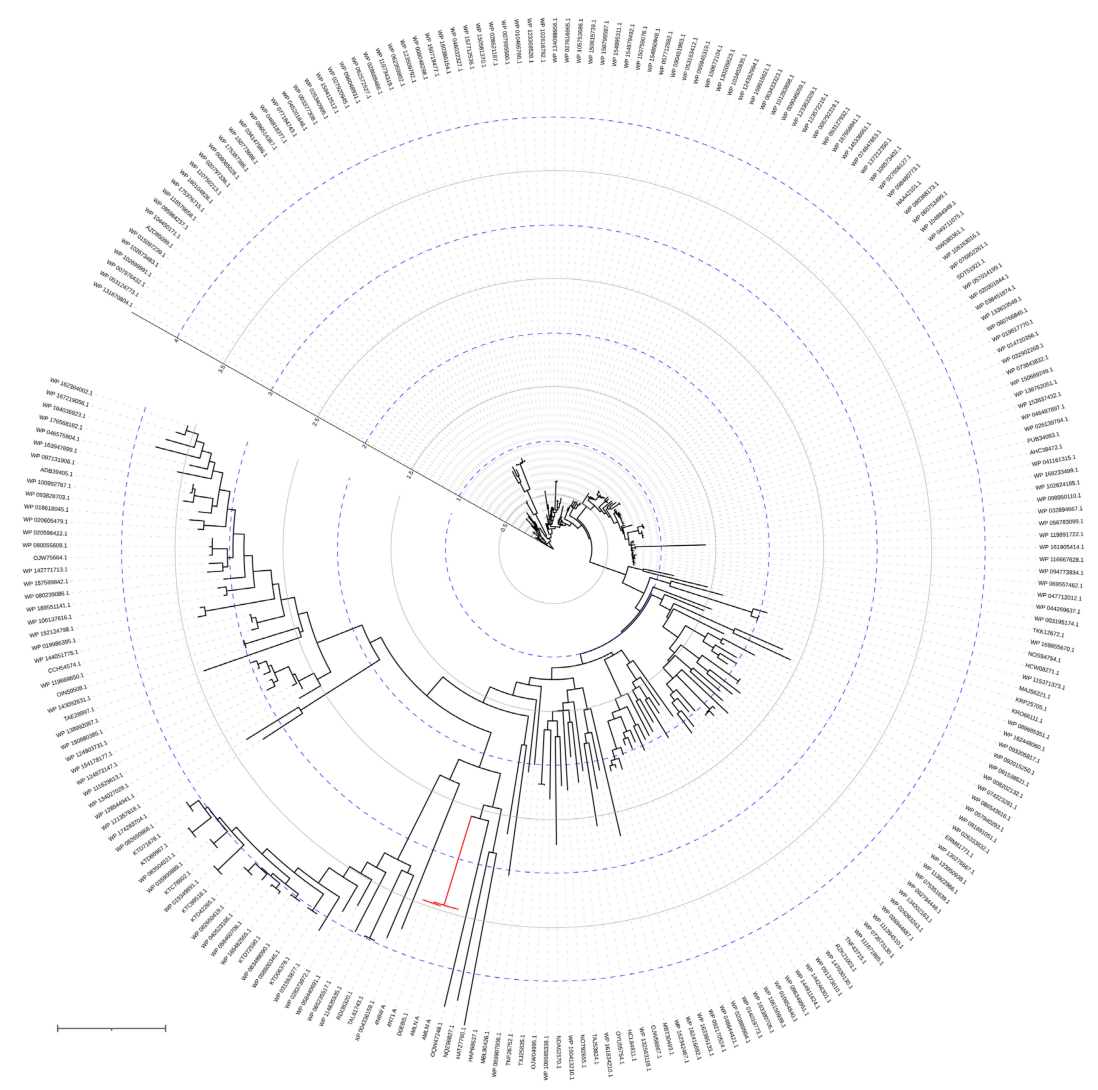
Phylogenetic tree of PhnZ and its homologous enzymes (down to 44% identity). The tree was built as previously with PDB 4MLN as a reference sequence. Enzymes having high (90–100%) identity are highlighted with red.

**Scheme 12:**
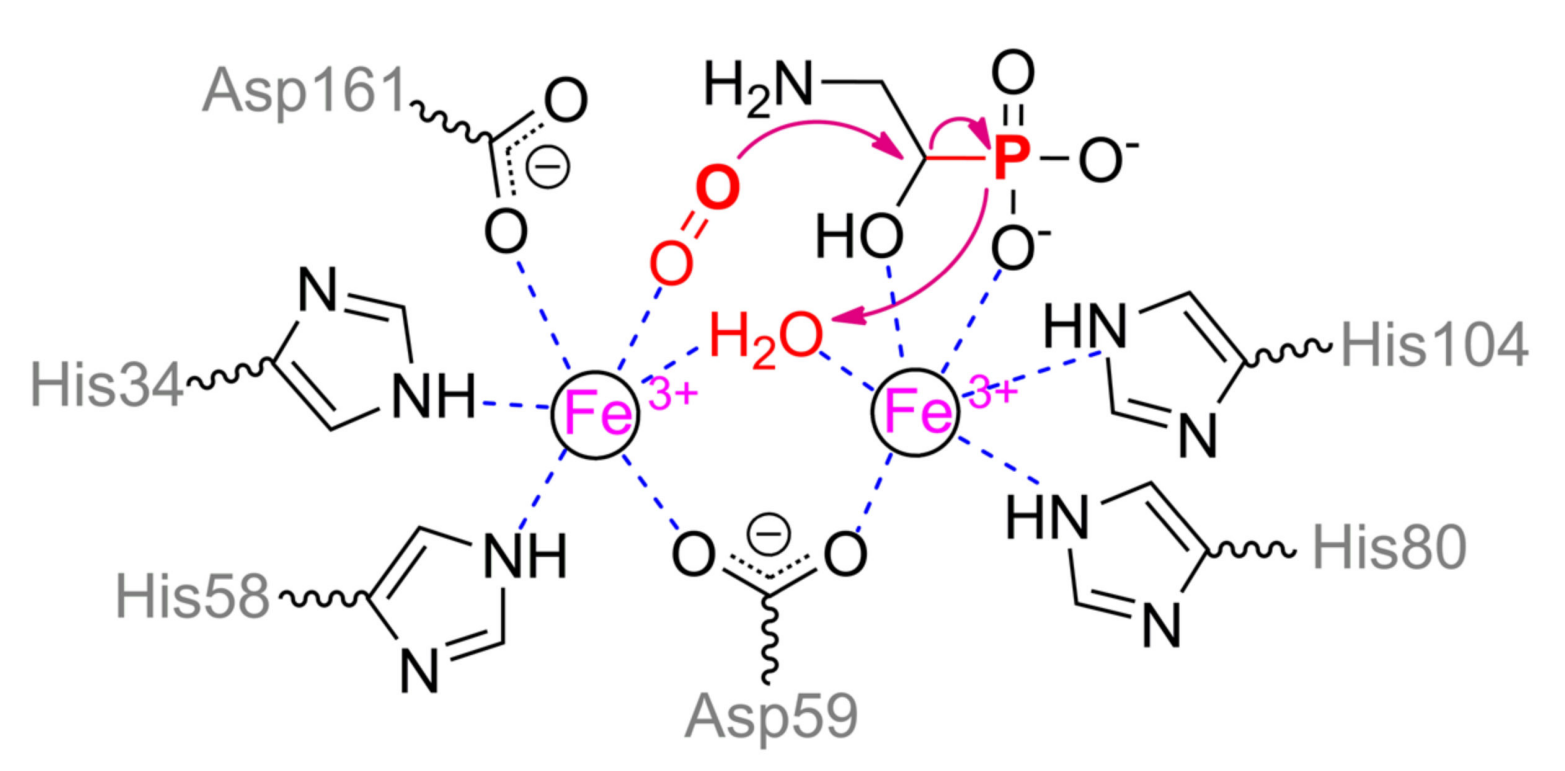
Mechanism of catalytic action of PhnZ.

Above-mentioned 2-aminoethylphosphonate can be metabolized in another pathway also [95]. At the first step, nonheme iron/α-ketoglutarate dependent dioxygenase PhnY hydroxylates this compound at α-carbon. Further (*R*)-2-amino-1-hydroxyethylphosphonate is cleaved by PhnZ, yielding glycine and phosphate. PhnZ relates to the diverse HD domain superfamily of enzymes and is rapidly evolving that results in a dramatic drop of identity from 97% to less than 60% (Figure 20). Anyway, it conserves a number of catalytically and structurally important features. PhnZ belongs to oxygenases and contains two iron ions of mixed valence Fe(II)/Fe(III) that are crucial for catalytic function [96]. The more buried α-metal ion is fully oxidized and coordinated by His80 and His104 (Scheme 12). Slightly more exposed β-metal ion is initially reduced and coordinated by His34, His58, Asp161, and water molecule (or Tyr24). Both metals are coupled via Asp59 and hydroxyl ion (or water molecule). Such hydroxyl anion could be formed by proton transfer in a chain Asp161–Arg158–Glu162. The substrate binds to α-metal via phosphonyl and hydroxyl oxygens while co-substrate (dioxygen) replaces water molecule of β-metal, and thus both metals have an octahedral coordination sphere. Proper substrate orientation is provided by interaction of amino group with Glu27 and of the hydroxyl group with His62, and modification of the substrate or enzyme residue(s) results in substantial activity decreasing [97].

Binding of co-substrate leads to oxidation of β-metal ion and generation of peroxide radical. *Myo*-inositol oxygenase has the same structure of the active center that allows the proposal of analogous mechanism of action for PhnZ. Initially, superoxide radical abstracts hydrogen from α-carbon atom [98] but its rotation towards bridging hydroxyl ion leads to a backward attack of peroxide oxygen by this carbocation and C–O bond formation followed by peroxide bond cleavage. A large aggregate negative charge stimulates this proton to migrate back and to coordinate with both oxygens. However, it is insufficient to stabilize this system and proton resides with ferryl moiety, forming Fe(III)OH. An interesting question here is the regeneration of initial enzyme state of PhnZ and the fate of C–P bond. The C–C bond within *myo*-inositol is cleaved while producing carboxy group (in the site of radical attack) and aldehyde (in the counterpart). It means that another carbon suffers a single electron oxidation and that should be the route for phosphorous also. The formal charge of phosphorous (and thus its oxidation state) within 2-amino-1-hydroxyethylphosphonate is ca. +4.22 according to QM calculations with B3LYP/6-311G/NPA forcefield in AtomicChargeCalculator (http://webchem.ncbr.muni.cz/Platform/ChargeCalculator) [99]. However, the formation of metaphosphoric acid proposed in [95] and repeated in [86] is unlikely since a direct donation of the bridging hydroxyl is available. Firstly, it will finish the cycle while transferring the electron to the β-metal ion. Secondly, such uncoupling is visible in the ‘intermediate state’ by EPR spectroscopy [96]. Later, another water molecule can diffuse from a solvent and recover the active state.

**Figure 21:**
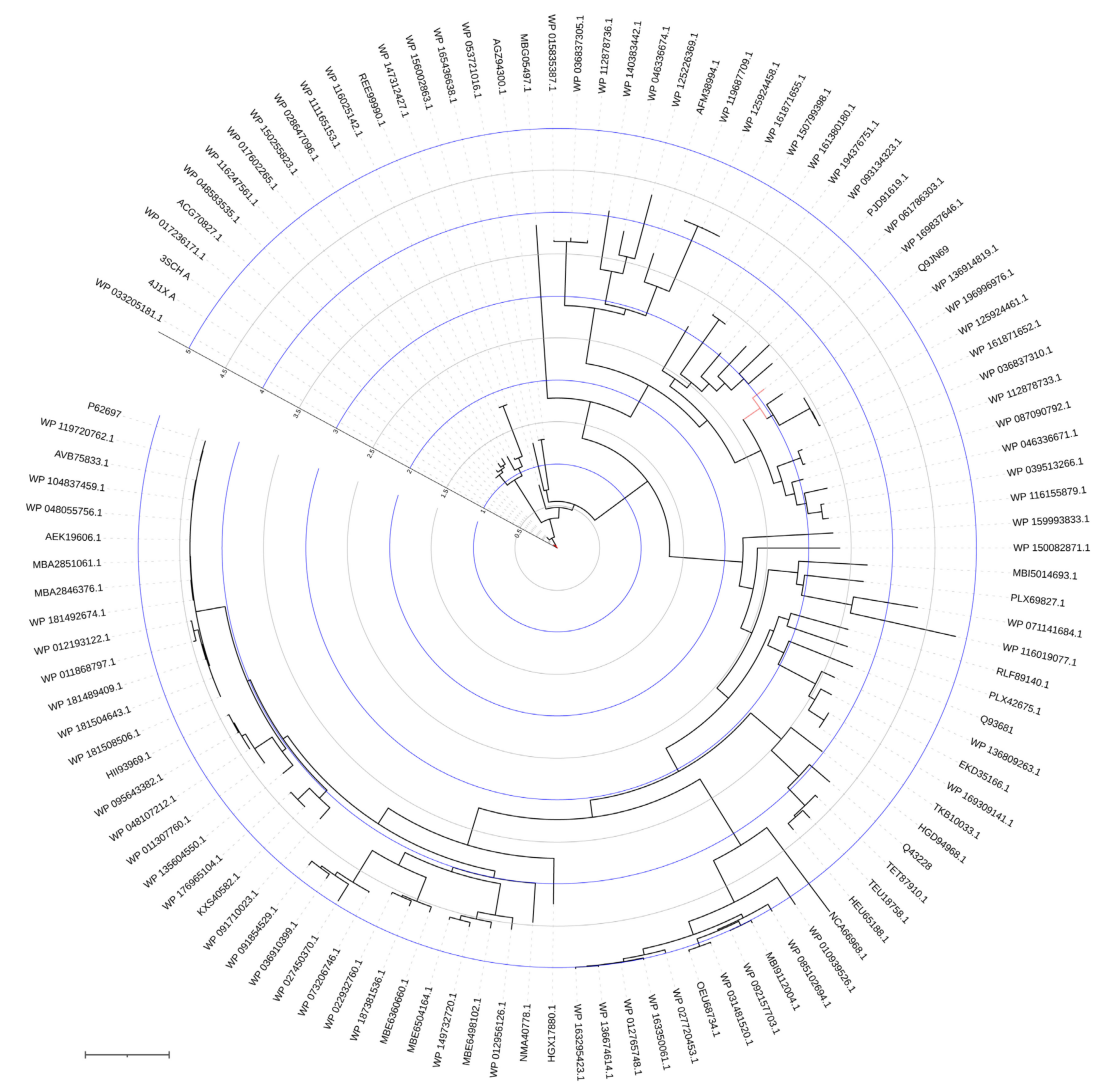
Phylogenetic tree of HppE and its homologous enzymes (down to 25% identity). The tree was built as previously with PDB 5U58 and 3SCF as reference sequences. Enzymes having high (90–100%) identity with 5U58 and 3SCF are highlighted with red and pink, respectively.

**Scheme 13:**
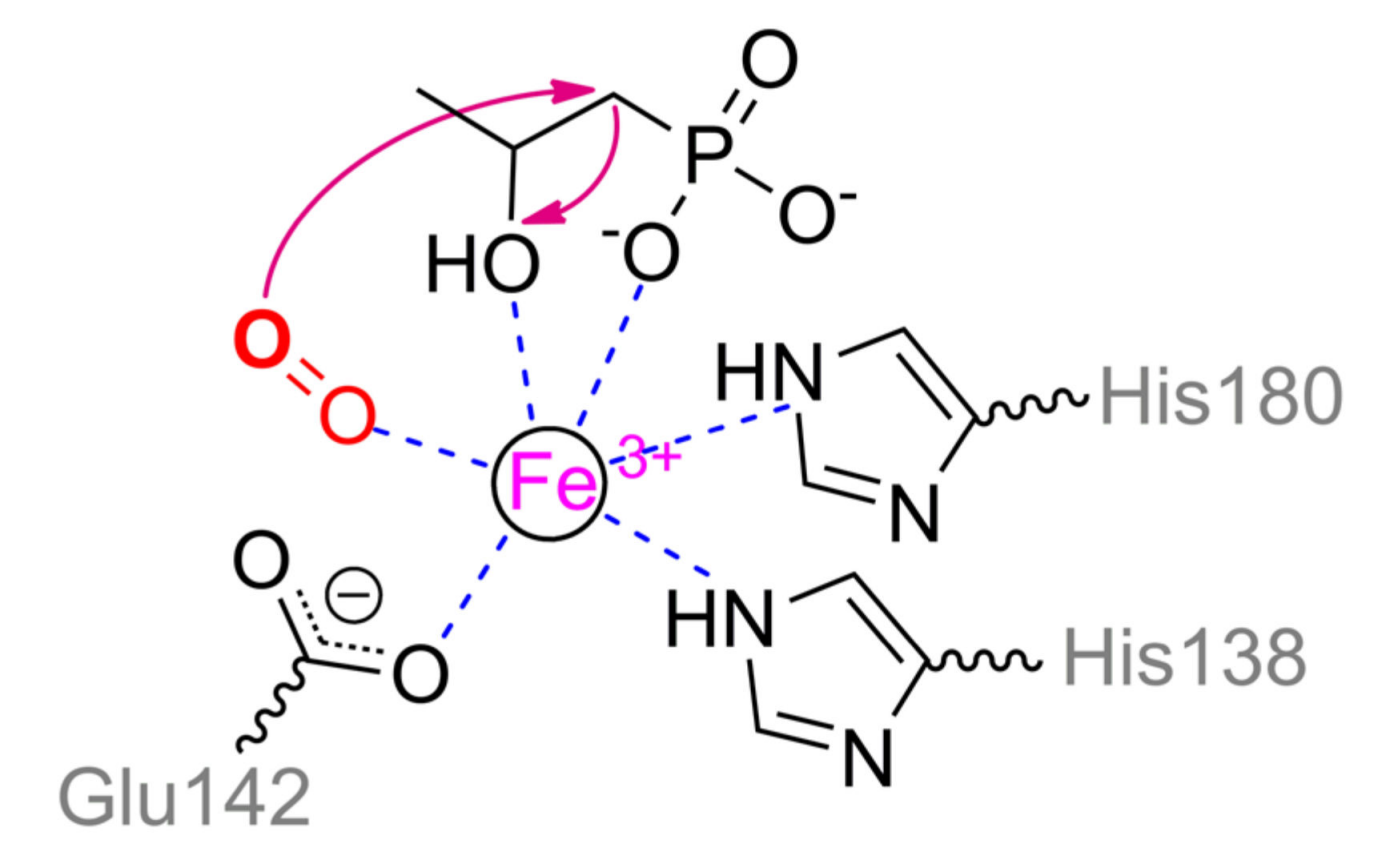
Mechanism of catalytic action of HppE.

Interestingly, 2-hydroxypropylphosphonic acid epoxidase (HppE) implements partially similar biochemical mechanics as PhnZ. An enzyme from *Streptomyces wedmorensis* cells is the most investigated and located basally (Figure 21) though other homologs, for example, evolutionally novel enzyme from *Pseudomonas syringae* (26% identity), are also issued [100]. HppE forms homotetramer and naturally participates in fosfomycin (i.e., natural antibiotic with phosphonate moiety) biosynthesis [86]. However, it contains a single Fe^2+^ ion and thus substrate transformation is limited while C–P bond is not cleaved in the end-product at all. Nevertheless, temporal cleavage of this bond is possible in the intermediate state with a number of substrates [101]. The single metal ion is coordinated by His138, Glu142, and His180 in enzyme from *S. wedmorensi*or or by His128, Glu132, and His171 in enzyme from *P. syringae*. (*S*)-2-Hydroxypropylphosphonic acid binds via phosphonyl and hydroxyl oxygens while co-substrate (dioxygen) completes an octahedral coordination sphere (Scheme 13). As in the case of PhnZ, co-substrate oxidizes metal and generates peroxide radical which abstracts the closest hydrogen from carbon. Depending on the substrate structure, it can be α- or β-carbon [102]. Carbocation is generated that results in intermolecular cyclization (in the case of natural substrate) or in structural rearrangements including a temporal C–P bond cleavage. Reaction path mainly depends on ionization energy of relevant carbon that by-turn is predetermined by its substituent(s) [101]. Finally, a single electron and hydrogen atom should migrate back to Fe(III)OOH, thus, reducing a metal ion and regenerating an initial enzyme state. It should be noted also that HppE converts 2-amino-(*R*)-1-hydroxypropylphosphonic acid (i.e., analogue of PhnZ substrate) into 2-keto-(*R*)-1-hydroxypropylphosphonate via a hypothetical imino-derivative [102] though the pathway of PhnZ (with peroxide attack) cannot be excluded.

**Figure 22:**
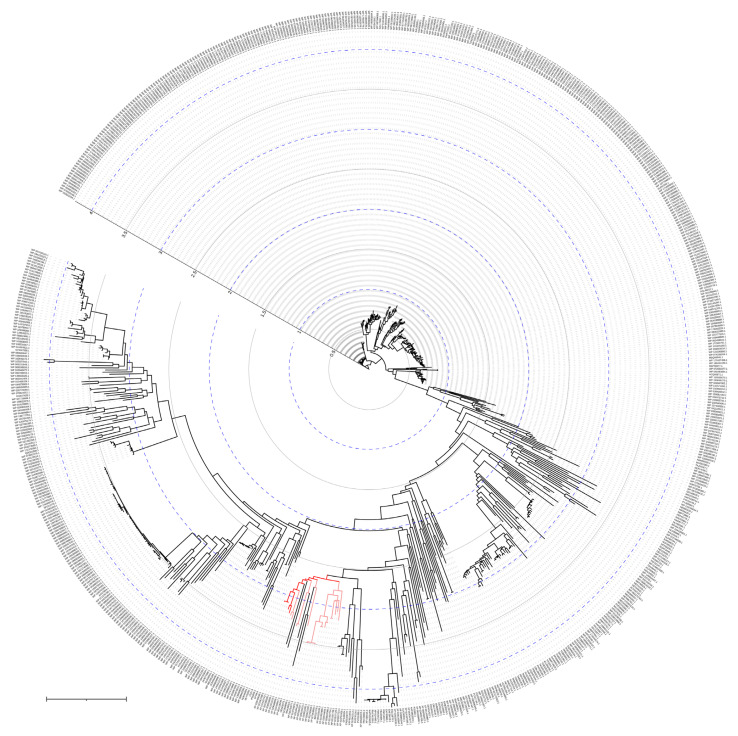
Phylogenetic tree of PEPP and its homologous enzymes (down to 64% identity). The tree was built as previously with PDB 1S2V as a reference sequence. Enzymes having high (90–100%) and good (80–89%) identity are highlighted with red and pink, respectively.

**Figure 23:**
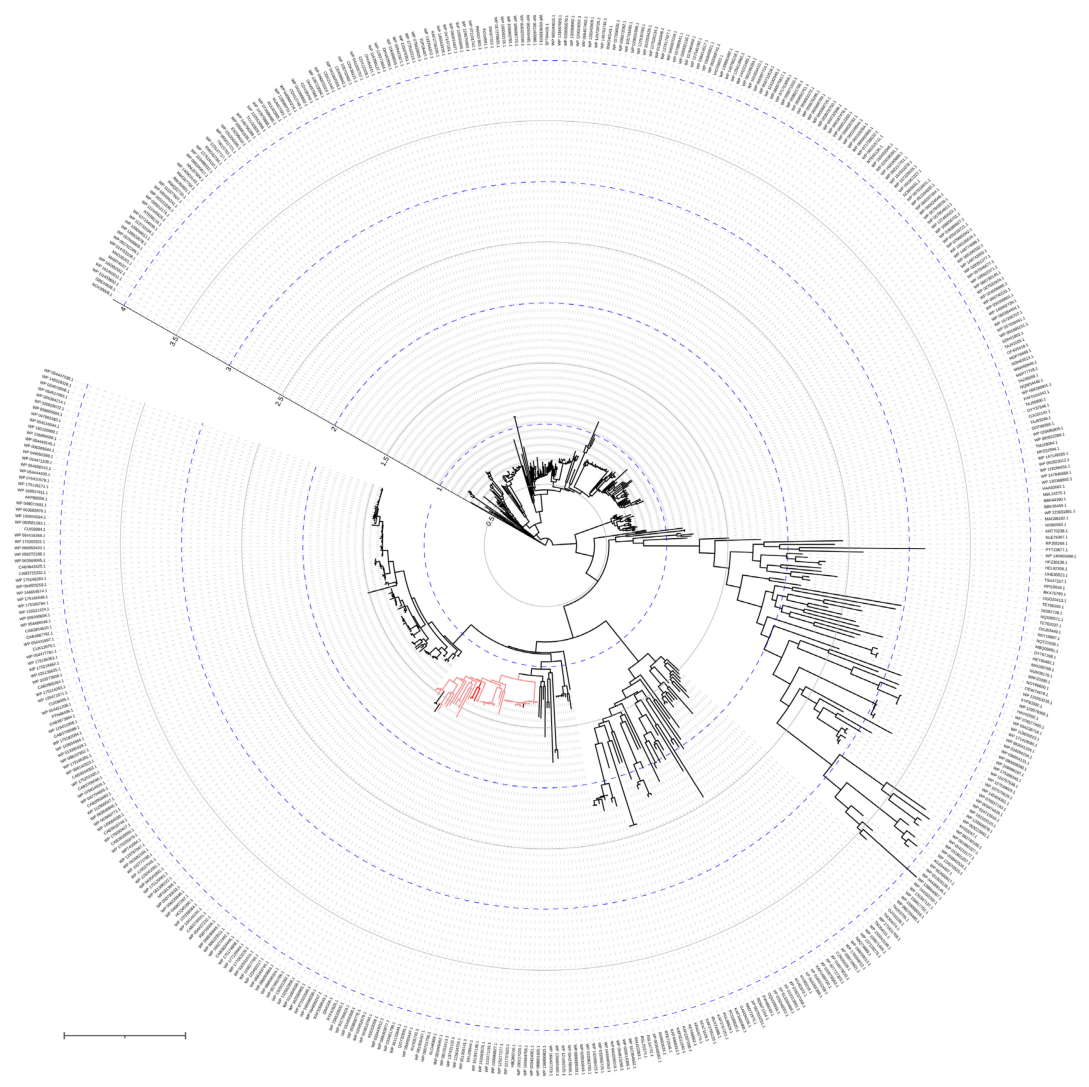
Phylogenetic tree of PPH and its homologous enzymes (down to 45% identity). The tree was built as previously with PDB 2HJP as a reference sequence. Enzymes having high (90–100%) and good (80–89%) identity are highlighted with red and pink, respectively.

**Scheme 14:**
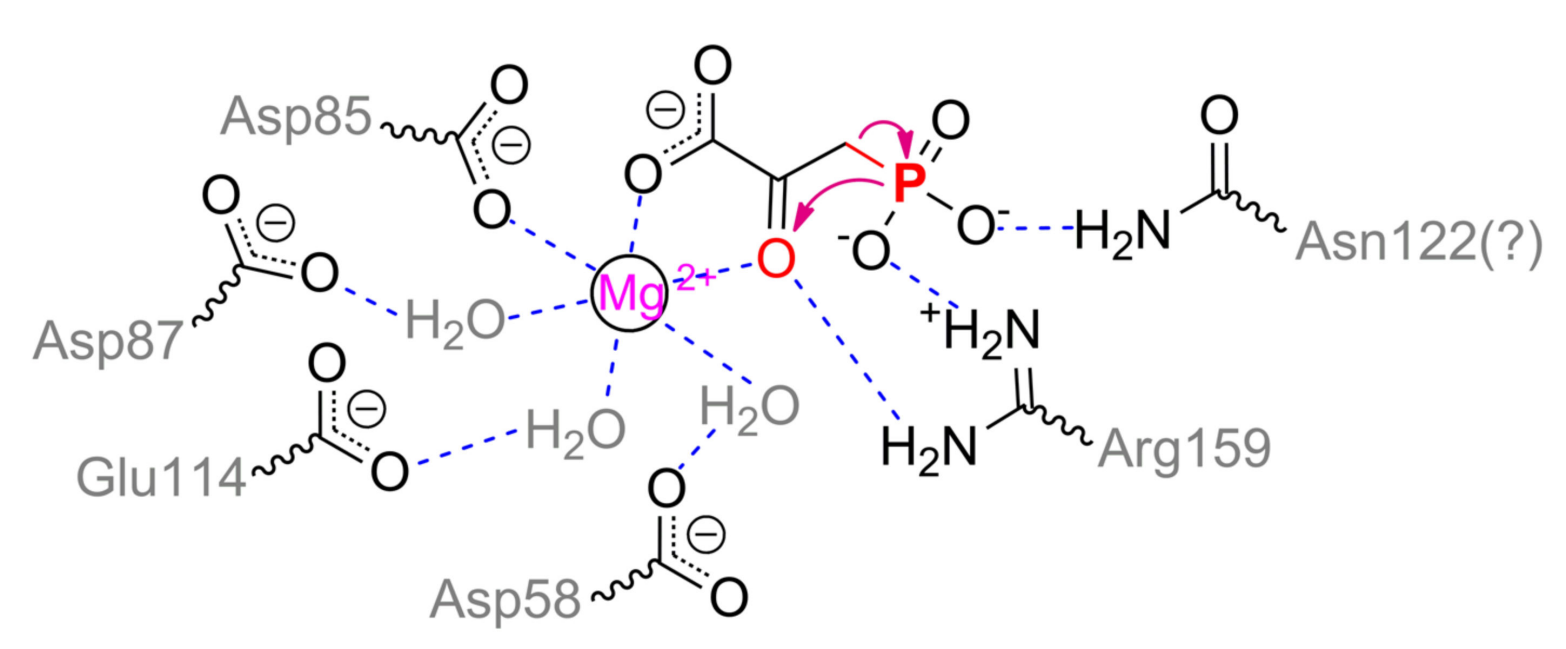
Mechanism of catalytic action of PEPP.

The above-mentioned keto-derivative is quite close to a phosphonopyruvate, being an entrance substance for almost all biologically produced phosphonates [86]. This phosphonopyruvate is a substrate/product of another interesting enzyme, namely, phosphonopyruvate hydrolase (PPH) and phosphoenolpyruvate phosphomutase (PEPP) (Figure 16b). Both of them belong to Phosphoenolpyruvate mutase/Isocitrate lyase-like family and have a TIM barrel fold of subunit though identity between them is 39%. Both enzymes are relatively novel and evolving (Figure 22 and Figure 23). Both of them natively contain a single Mg^2+^ ion, though PPH has better kinetic parameters with Co^2+^ [103]. PEPP forms a homodimer though there are plugs to assemble a tetramer. The metal ion in PEPP is octahedrally coordinated by Asp85 and five water molecules, three of which are hold by Asp58, Asp87, and Glu114 (Scheme 14). Two other water molecules are replaced by substrate bound bidentantly via carboxyl and keto oxygens (for phosphonopyruvate). Arg159 is located close enough to keto oxygen and provokes a distortion in the octahedral coordination sphere which is essential for catalysis [104]. Besides, it together with His190 (and hypothetically with Asn122 and Ser123, see below) is capable to interact with phosphonyl oxygens and thus stabilizes phosphorous intermediate. Such intermediate is most likely to be metaphosphate-like with uncompensated partial positive charge on the phosphorous atom and thus it is able to bind with keto oxygen and terminal methylene carbon [86]. Kinetically, an equilibrium is shifted towards the phosphoenolpyruvate formation though QM/MM simulations revealed a higher energy barrier in this path [105]. It seems some greater conformational changes are taking place during “open–closed” enzyme transition [106] and were neglected accidentally. Additionally, it is still unknown what is the real reason of C–P bond cleavage, for example, in phosphonopyruvate.

**Scheme 15:**
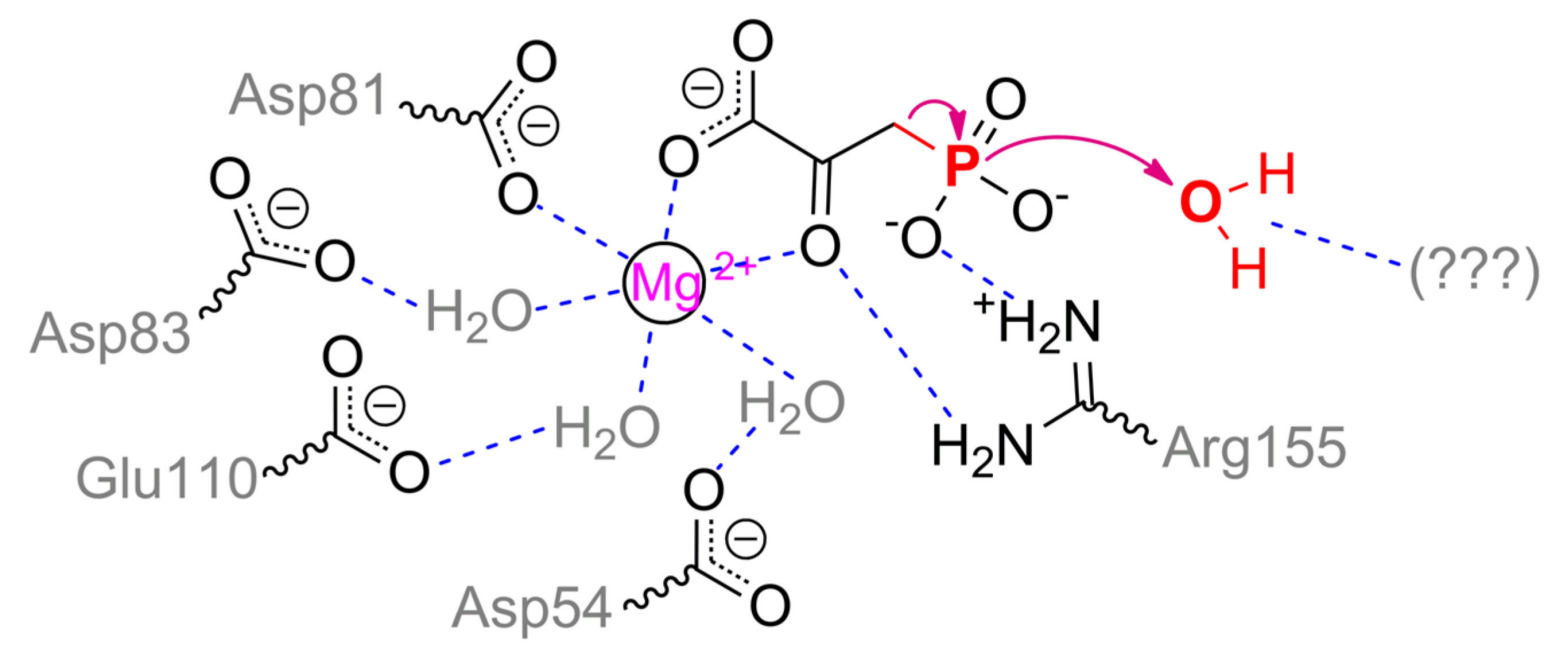
Mechanism of catalytic action of PPH.

As in PEPP, the metal ion in PPH is coordinated by Asp81, three water molecules bound to Asp54, Asp83, and Glu110, and by two waters lately replaced with substrate (Scheme 15). Phosphonyl oxygens are capable to interact with Arg155, His186, and Arg188 in open enzyme conformation, but in a closed form, Arg188 is likely to have an influence on a proper substrate binding only and to be insignificant in catalysis itself [103]. Thus, the main cause in the path of phosphonopyruvate transformation by PEPP and PPH (i.e., intramolecular transfer or complete hydrolysis) is a mobile loop/cap. Its mostly differing sequences are ^121^TNSLHDGRAQPLAD^134^ and ^117^DTSLRTDGRQELVR^130^ (identical residues are underlined) in PEPP and PPH, respectively. Most authors attribute catalytic differences of PEPP and PPH to a single substitution Asn122Thr and additional water molecule retention within closed enzyme form [86,103]. However, it is unlikely to be the real reason.

Though a total charge of both sequences is zero at neutral pH values, the absolute positive/negative charges in the loop of PPH are greater. Moreover, theoretical pI values for these sequences in PEPP and PPH are 5.2 and 6.1, respectively. The loop in PEPP is harder, but the same loop in PPH is more massive (by 10%) and has additional threonine residue. Both enzymes have similar pH profiles with p*K*_a_ of 7–7.5 and thus differing protonation/deprotonation within loop(s) is unlikely to explain the differences. Noteworthily, a triplet Asp–Gly–Arg is shifted in PPH and there is substitution Pro131Glu. As it was discussed previously, the best mechanics implemented naturally to activate water molecules are through acid/base catalysis. Therefore, the same mechanism could be in PPH with glutamate, arginine, and/or threonine. Further research could elucidate the precise path of phosphonopyruvate hydrolysis by PPH.

## 13. Concluding Remarks and Future Perspectives

A great number of enzymes studied to date and capable of detoxifying OPCs have diverse structures and mechanisms of catalytic action. The main attention was given to hydrolases which don’t require any special additives to the reaction medium for their functioning, and thereby hydrolases are the most widely represented among discussed enzymes.

Most of these hydrolases have a pH-optimum in the range from neutral to slightly alkaline pH values. Products formed as a result of OPCs hydrolysis have a much lower toxicity as compared to initial substances, and thus, a detoxifying effect is achieved. Moreover, even complete sequential degradation of OPCs is possible [12] in a series: triester→ diester → monoester → phosphate; or in any other cascades [107]. All enzymes have their own differing catalytic specificity though their substrate range may overlap significantly not only with OPCs but with other ‘natural’ substrates, such as activity of discussed PLL-like enzymes, OPH, PON, etc. with *N*-acyl homoserine lactones [108,109]. Thus, they can be rationally combined to obtain universal enzyme preparations [110] or even hybrid molecules with other enzymes [111] or with ‘guide’ DNA [112]. This opens up new promising perspectives for their practical application.

Though many enzymes being active with OPCs were purified and characterized to date, as it was shown in the current review this is only a tip of the true diversity of available enzymes. As in the case of PEPP and PPH, their catalytic mechanism of action can be shifted dramatically via simultaneous structural modifications. It is reasonable to assume that future researches could greatly expand our simplistic mechanistic understanding which was presented here.

Another point is a fine organic synthesis. Though the industry didn’t show an urgent interest in it for decades, the current epidemiological situation could change something because there are a lot of OPCs of medical importance [113].

At the same time, the widespread implementation of computer modeling allows not only to explain the already obtained experimental data but also to generate the necessary scientific basis for the rational selection of such catalytically effective combinations of enzymes that seem to be the most valuable for further practical use.

## Abbreviations

AChE	Acetylcholinesterase
ACP	Acid phosphatase
ALP	Alkaline phosphatase
AMPP	Aminopeptidase P
DFPase	Diisopropyl-fluorophosphatase
DFT	Density functional theory
HppE	2-Hydroxypropylphosphonic acid epoxidase
MPH	Methyl parathion hydrolase
OPAA	Organophosphorus acid anhydrolase
OPC	Organophosphorus compound
OPH	Organophosphorus hydrolase (=phosphotriesterase)
PEPP	Phosphoenolpyruvate phosphomutase (=PEP mutase)
PhnA	Phosphonoacetate hydrolase
PhnJ	C–P lyase subunit J
PhnX	Phosphonoacetaldehyde hydrolase (=phosphonatase)
PhnZ	(*R*)-2-Amino-1-hydroxyethylphosphonate oxygenase
PLL	Phosphotriesterase-like lactonase
PON1	Paraoxonase 1
PPH	Phosphonopyruvate hydrolase
QM/MM	Combined quantum-mechanical / molecular-mechanical [modeling]
SMP30	Senescence Marker Protein 30

## Appendix A

Enzyme–substrate complexes were modeled as described previously [68]. Briefly, structures of OPCs were prepared and docked to enzymes at certain pHs using AutoDock Vina (ver. 1.1.2, available at http://vina.scripps.edu/) [114] on a desktop computer. The most reliable poses were visualized with PyMOL Molecular Graphics System (ver. 1.7.6, Schrödinger, LLC, New York, NY, USA).

To build a phylogenetic tree, initially database of non-redundant protein sequences was searched for homologues to a certain enzyme by blastp algorithm (available at https://blast.ncbi.nlm.nih.gov/Blast.cgi) at default options. To expand sampling, organisms containing the highest rated homologue(s) were iteratively excluded from the search. Then, search results were combined and multiply aligned with Clustal Ω (available at https://www.ebi.ac.uk/Tools/msa/clustalo/) at default options. The tree was generated using phylogenetic likelihood library [115] with IQ-TREE (available at http://iqtree.cibiv.univie.ac.at/) at default options and visualized with iTOL (available at https://itol.embl.de/).
